# Chemogenetic ubiquitin E1 inhibitor TAK-243 CRISPR screen uncovers HAPSTR1/HUWE1 axis in chromatin proteostasis and cell cycle control

**DOI:** 10.1126/sciadv.aea3388

**Published:** 2026-10-16

**Authors:** Nila K. van Overbeek, Linda Clijsters, Martina Wirth, Ilona J. de Graaf, Klaas de Lint, Sidrit Uruci, Amina F. A. S. Teunisse, Vyacheslav Akimov, Arnoud H. de Ru, Maja Vukic, Idil Kirdök, Lucia Daxinger, Peter A. van Veelen, Blagoy Blagoev, Martijn S. Luijsterburg, Rob M. F. Wolthuis, Ilana Berlin, Alfred C. O. Vertegaal

**Affiliations:** ^1^ Department of Cell and Chemical Biology, Leiden University Medical Center, Leiden, Netherlands.; ^2^ Department of Human Genetics, Amsterdam University Medical Center, Amsterdam, Netherlands.; ^3^ Cancer Center Amsterdam, Amsterdam University Medical Center, Amsterdam, Netherlands.; ^4^ Department of Human Genetics, Leiden University Medical Center, Leiden, Netherlands.; ^5^ Department of Biochemistry and Molecular Biology, University of Southern Denmark, Odense, Denmark.; ^6^ Center for Proteomics and Metabolomics, Leiden University Medical Center, Leiden, Netherlands.; ^7^ Oncode Institute, Leiden University Medical Center, Leiden, Netherlands.

## Abstract

In this study, we sought to comprehensively map ubiquitin signaling networks at the crossroads of cellular proliferation and proteostasis, both crucial in cancer. Genome-wide CRISPR knockout screening in the presence of the ubiquitin E1 inhibitor TAK-243 identified 239 genes whose ablation sensitizes cells to ubiquitination blockade. Follow-up studies reveal a role for the nuclear protein, C16orf72/HECT, UBA, and WWE domain containing E3 ubiquitin protein ligase 1 (HUWE1)–Associated Protein modifying Stress Responses (HAPSTR1), in chromatin proteostasis and DNA replication. We show that HAPSTR1 recruits the E3 ligase HUWE1 to the chromatin, keeping the chromatin-associated proteome in check, and is itself turned over by HUWE1 during cell cycle progression. Structural modeling of the HAPSTR1/HUWE1 complex, supported by mutational analysis, demonstrates that loss of HUWE1 binding renders HAPSTR1 unable to support cellular proliferation when ubiquitin signaling is suppressed. Consequently, HAPSTR1 ablation impairs clearance of chromatin-associated proteins, triggering global transcriptional and proteostatic adaptations. Under diminished ubiquitin signaling, these adaptive mechanisms give way to replication stress and premature S phase entry, culminating in cell cycle collapse and DNA damage. Our findings uncover protein networks exhibiting synthetic lethality with ubiquitin signaling blockade and highlight vulnerabilities of actively proliferating cells to proteostatic imbalance at the chromatin.

## INTRODUCTION

Controlled cellular proliferation underlies development and tissue regeneration, while uncontrolled proliferation is associated with a broad spectrum of pathologies, notably including cancer (*1*). Proliferation initiation hinges on the decision to enter the cell cycle (G_0_/G_1_ phase), which licenses duplication of the genome (S phase), followed by a secondary gap period (G_2_ phase) in preparation for mitosis. During mitosis (M phase), condensation, alignment, and segregation of chromosomes, as well as partitioning of organelles and the soluble proteome, take place, ultimately resulting in the birth of daughter cells (*2*). To safeguard fidelity of cellular proliferation, multiple checkpoints are enforced across the cell cycle. DNA damage triggers these cell cycle checkpoints to allow timely repair and thus maintains cell cycle fidelity, whereas loss of DNA damage checkpoint integrity contributes to cancer development (*3*).

Unidirectional transitions between the cell cycle phases are orchestrated by a complex network of reversible posttranslational modifications (PTMs) that enable protein activity, stability and localization to be dynamically controlled. Chemical PTMs such as phosphorylation and modifications with small proteins, including ubiquitin and SUMO, play key roles in cell cycle progression and genome stability (*4*–*6*). While phosphorylation is added in a single reaction step, catalyzed by kinases and reversed by phosphatases (*7*), covalent attachment of ubiquitin and ubiquitin-like modifiers typically requires a cascade of activating E1, conjugating E2, and ligating E3 enzymes (*8*, *9*). Subsequently, deubiquitinating enzymes (DUBs) can remove ubiquitin from cellular substrates, rendering ubiquitination dynamic (*10*). Transitions between cell cycle phases are driven by waves of activation and inhibition of cyclin-dependent kinases (CDKs) through their cyclin coactivators and CDK inhibitors (CDKIs). Regulation of cyclin abundance is largely controlled at the level of proteostasis, i.e., the balance between protein synthesis and degradation. The latter is orchestrated by the ubiquitin proteasome system (UPS) (*9*), wherein ubiquitination serves to label proteins for on demand destruction and thus enables precise timing of cell cycle events (*11*). Although ubiquitin signaling cascades are capable of shifting modification profiles at the proteome-wide level in mere minutes (*12*, *13*), the global impact of ubiquitination on protein networks governing cell cycle progression remains elusive.

In this study, we sought to identify genes required for ubiquitin signaling and proteostasis in the context of cell proliferation and cell cycle control. To accomplish this, we devised a chemical-genetic CRISPR knockout (KO) screen exploiting the first-in-class ubiquitin E1 inhibitor TAK-243 (*14*) as a tool to instigate global ubiquitin blockade. Using this methodology, we identified gene networks associated with sensitization or resistance to diminished ubiquitin signaling, providing a genome-wide overview of the interplay between the ubiquitin system and cell proliferation. Our dataset uncovers a synthetic lethal interaction between C16orf72/HECT, UBA, and WWE domain containing E3 ubiquitin protein ligase 1 (HUWE1)–Associated Protein modifying Stress Responses (*HAPSTR1*) gene disruption and ubiquitin blockade. Mechanistically, we describe a role for HAPSTR1 in chromatin-associated proteostasis through recruitment of the ubiquitin ligase HUWE1 to chromatin, which is maintained under dynamic control by cell cycle dependent degradation of HAPSTR1 by the UPS. In the absence of HAPSTR1, cells exhibit impaired replication fidelity and aberrant cell cycle progression, evoking adaptive rewiring of the transcriptome and ubiquitinome to maintain cell survival.

## RESULTS

### Regulators of ubiquitin signaling are important for cell proliferation

To identify regulators of ubiquitin signaling required for cell proliferation, we used the ubiquitin E1 inhibitor TAK-243 (Fig. 1A and fig. S1A) in a chemical-genetic CRISPR-Cas9 KO screen (Fig. 1B). The TKOv3 lentiviral library (*15*), consisting of up to four single guide RNAs (sgRNAs) per gene targeting 18,053 protein coding genes, was introduced into RPE1-p53^−/−^ cells expressing doxycycline-inducible Cas9 (fig. S1B) to screen for genes that alter sensitivity to TAK-243. Initially, the administered dosage of 0.1 μM TAK-243 was chosen to reduce but not abolish ubiquitin signaling in RPE1-p53^−/−^ cells, and treatment regimen was gradually increased up to 0.4 μM TAK-243 to prevent habituation (fig. S1, C and D). Deep sequencing conducted after 12 population doublings in the presence of TAK-243 identified 239 genes whose ablation renders cells sensitive to diminished ubiquitin signaling, as well as 55 genes whose loss causes resistance toward TAK-243 (Fig. 1C and data S1). In line with a prior report on sensitization of acute myeloid leukemia (AML) cells (*16*), the adenosine 5′-triphosphate (ATP)–binding cassette drug transporter ABCG2 (*17*) ranked as the top synthetic-lethal gene in our screen (Fig. 1C and fig. S1E), implying that ABCG2 mediates TAK-243 efflux.

**Fig. 1.**
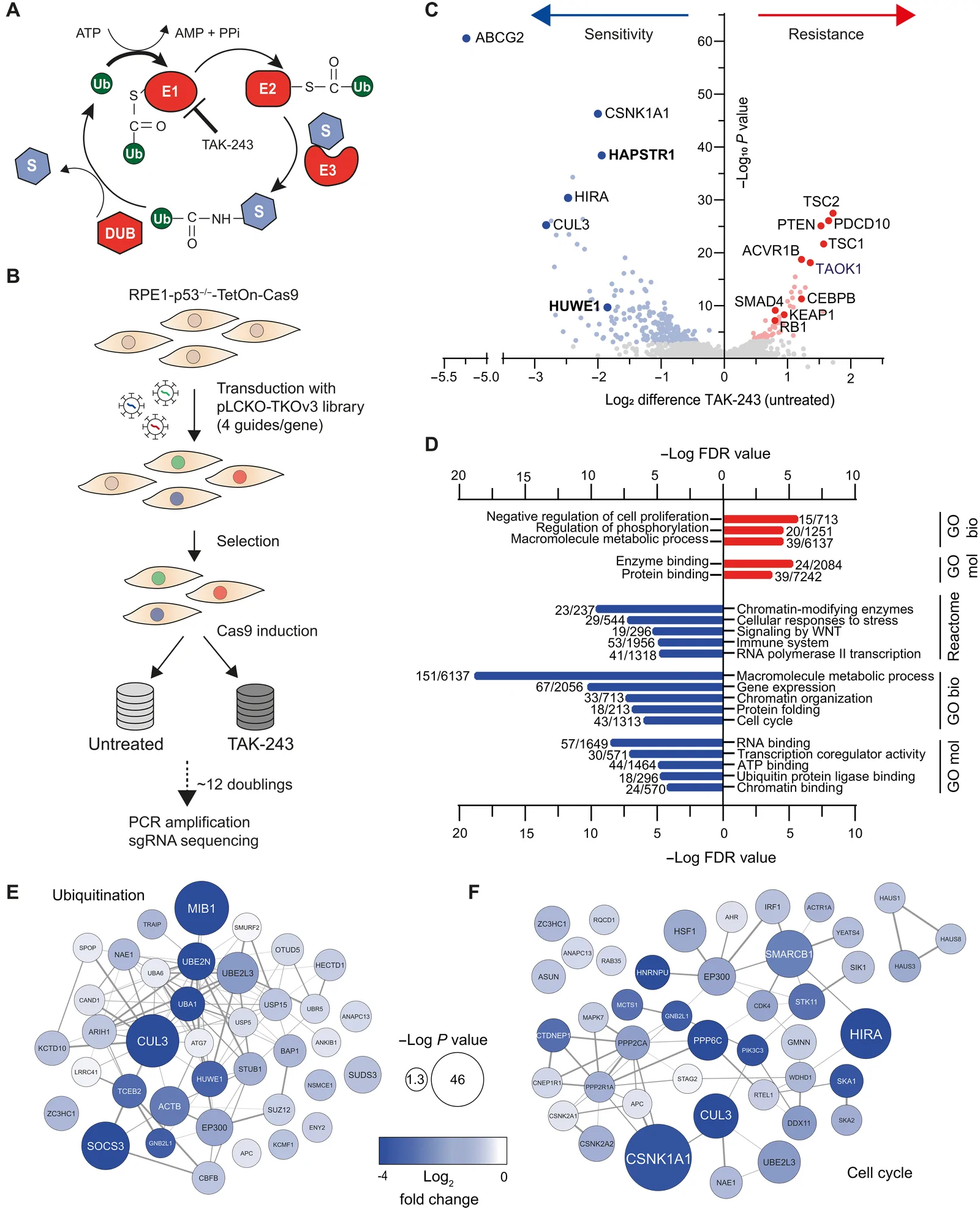
Chemogenetic CRISPR KO screen reveals critical regulators within the ubiquitin signaling network essential for cell proliferation. (**A**) Schematic of the ubiquitin (Ub) conjugation cascade. Ub is conjugated to substrate (S) proteins via an enzymatic cascade of E1, E2, and E3 enzymatic activities and removed by DUBs. TAK-243 inhibits the E1 enzyme, thereby blocking Ub conjugation. (**B** to **F**) Chemogenetic CRISPR KO screen with TAK-243. (B) Workflow overview. RPE1-hTERT-p53^−/−^-PAC^−/−^-TetOnCas9 cells transduced with TKOv3 lentiviral library (*15*) and cultured with doxycycline to induce Cas9 expression. Cells were treated, or not, with TAK-243 (gradually increased from 0.1 to 0.4 μM over 12 days to prevent habituation) for 12 doublings and harvested, and guide RNAs (gRNAs) were PCR amplified and sequenced. (C) Volcano plot of gene hits whose loss sensitizes (blue) or confers resistance (red) to TAK-243, based on a false discovery rate (FDR) cutoff of 0.1. Log_2_ fold change and –log_10_ *P* value. Individual dots represent combined (four guides) normZ value per gene. Representative data from *n* = 3 independent experiments are shown. (D) Enrichment analysis of genes whose KO was significantly (FDR < 0.1) associated with resistance (red) or sensitization (blue) of cells to TAK-243. Shown are functionally enriched Reactome, Gene Ontology (GO) biological process and GO molecular function terms, and number of genes. STRING analysis subclusters of sensitizing genes involved in (E) protein ubiquitination and (F) cell cycle are shown. Circle size: –log *P* value; color intensity: log_2_ fold change; edge width: protein-protein interaction score. See also figs. S1 and S2 and data S1.

STRING analysis of hits from our screen revealed a core interconnected network comprising 221 out of 239 sensitizing genes, as well as a core network including 37 out of 55 genes conferring resistance (fig. S2A). Within these networks, Molecular Complex Detection (MCODE) assembled 11 and 2 subclusters for sensitization and resistance, respectively (fig. S2B). Further functional enrichment analysis indicated that genes required for TAK-243 tolerance are connected to crucial cellular processes such as gene expression, chromatin organization and stress response (Fig. 1D). Top resistance-associated genes included known negative regulators of cell proliferation, among which were TSC1, TSC2, PTEN, ACVR1B, and SMAD4 (Fig. 1C). Our screening data also unveiled a subset of ubiquitin conjugation machinery involved in cellular proliferation (Fig. 1E) and cell cycle regulators critical for proliferation under ubiquitin blockade (Fig. 1F). For instance, loss of either DNA replication regulator geminin (*18*) or phosphatase PPP6C involved in chromosome segregation (*19*), appears more challenging for cells with compromised ability to dynamically modulate their proteome through the UPS. Collectively, these findings provide a valuable resource revealing interplay between ubiquitin signaling, proteostasis and cellular proliferation.

Among the genes whose ablation greatly sensitized RPE1-p53^−/−^ cells toward ubiquitin blockade, we identified HUWE1 and the enigmatic HAPSTR1 (Fig. 1C) (*20*, *21*). Cells harboring sgRNAs targeting either HAPSTR1 or HUWE1 (but not another HECT E3 ligase NEDD4) were strongly selected against in the presence of TAK-243 (Fig. 2A and fig. S3A). To functionally validate the screen results obtained for HAPSTR1, we performed a multicolor competitive growth assay (MCA), where RPE1-p53^−/−^ cells lacking HAPSTR1 [green fluorescent protein^+^ (GFP^+^)] were mixed with control cells (mCherry^+^) and GFP/mCherry cell ratio was monitored over time (Fig. 2B). As expected, ubiquitin signaling suppression upon treatment with TAK-243 caused HAPSTR1-KO cells to be outgrown (Fig. 2C). Curiously, when HAPSTR1 abundance was evaluated by immunoblotting, sgRNA1 and sgRNA2 were found to not decrease protein levels, in contrast to sgRNA3 (fig. S3B). An in-depth analysis of gene editing efficiency and indel formation by different sgRNAs targeting HAPSTR1 revealed a 3–base pair deletion in roughly 60% of both sgRNA1- and sgRNA2-edited polyclonal cells (fig. S3C). Both sgRNAs introduced a single alanine deletion: sgRNA1 (ΔA69) and sgRNA2 (ΔA96) (table S1) positioned within a highly conserved region of HAPSTR1 (*22*), suggesting that mutations therein result in loss of function (Fig. 2D).

**Fig. 2.**
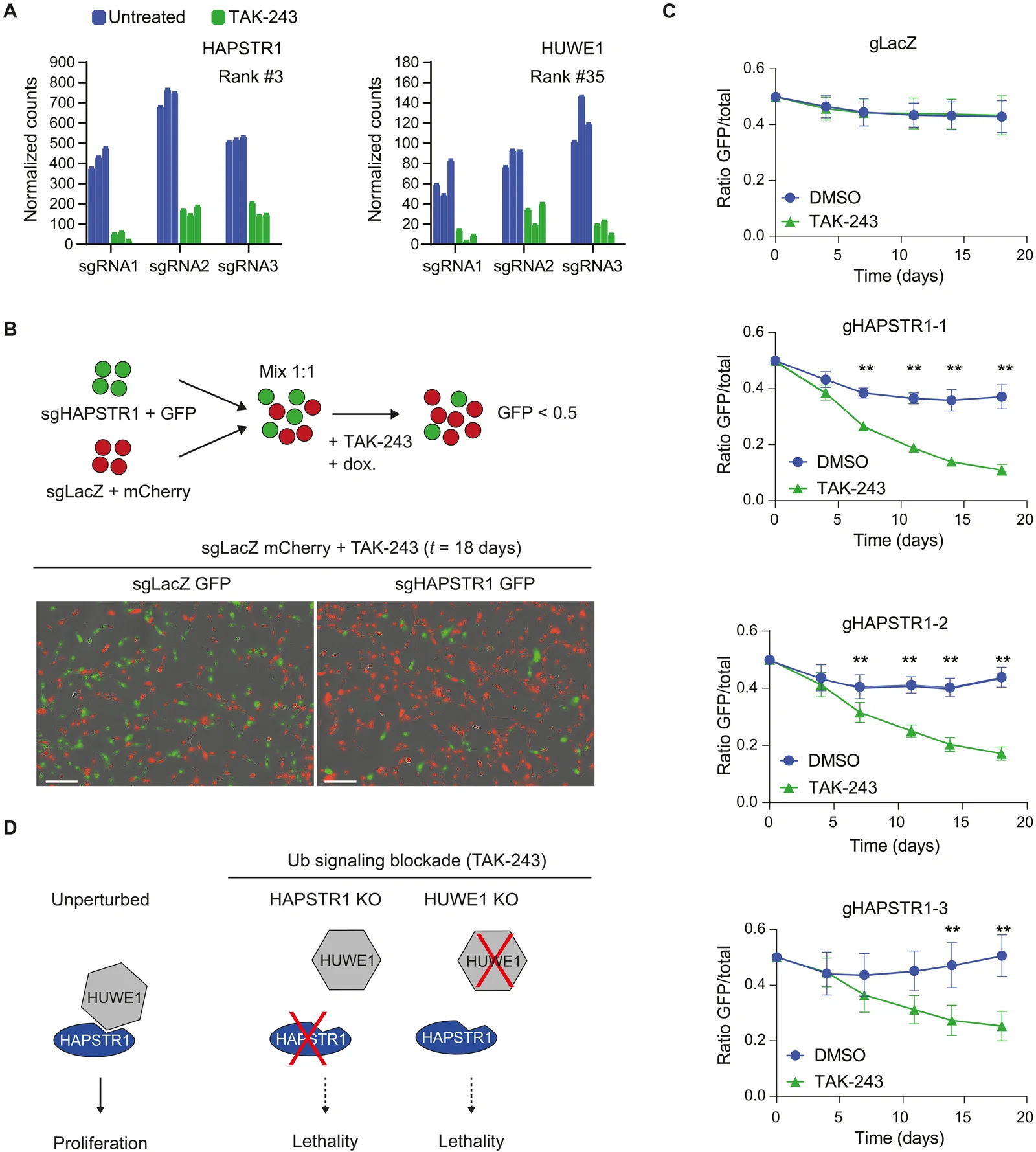
Ablation of HAPSTR1 sensitizes cells toward ubiquitin signaling blockade. (**A**) Sensitizing effect of HAPSTR1 and HUWE1 loss to TAK-243 treatment. Bar graph of normalized counts for individual sgRNAs targeting *HAPSTR1* or *HUWE1* genes isolated from RPE1-p53^−/−^ cells cultured in the presence (green) or absence (blue) of TAK-243. Plotted are *n* = 3 biological replicates from the chemogenetic screen in Fig. 1C. (**B** and **C**) MCA. RPE1-p53^−/−^ cells cotransduced with mCherry and control sgRNA (LacZ) were mixed with cells cotransduced with GFP and sgRNA targeting HAPSTR1 or LacZ (control). Cell growth was monitored by fluorescence imaging over time (days) following induce Cas9 induction (+dox.) in the presence or absence of TAK-243 (0.1 to 0.3 μM). Representative images at *t* = 18 days are shown. Scale bars, 200 μm. (C) Plots of GFP-positive cell fractions over time (days) from *n* = 3 independent experiments; mean ± SD, Mann-Whitney test, **P < 0.01. (**D**) Schematic of synthetic lethality of TAK-243 treatment with HAPSTR1 or HUWE1 ablation.

Given that loss of either HAPSTR1 or HUWE1 sensitizes cells to hampered ubiquitin signaling, we hypothesized that the interaction between the two proteins may be involved. HUWE1 is a large (4374 amino acids) solenoid-shaped E3 ligase (*23*) whose region spanning residues 2365 to 3090 was previously shown to mediate coimmunoprecipitation with HAPSTR1 (*20*). The same region is poorly resolved in existing structures (*20*, *23*), prompting us to model the HAPSTR1/HUWE1 binding interface using AlphaFold3. While initial modeling runs indicated widespread disorder (Fig. 3A), recurrent contacts within the C-terminal segment of HUWE1 prompted focused predictions using HUWE1 residues 3014 to 3049, which yielded a convergent, high-confidence interface [Fig. 3B; best interchain predicted TM score (ipTM) = 0.83]. Docking the model onto full-length HUWE1 [Protein Data Bank (PDB) 7JQ9] based on the tower motif suggests that HAPSTR1 binding is compatible with the overall HUWE1 architecture without steric clashes (Fig. 3C). The interaction is predicted to be mediated by a compact helix-based interface where a short α helix from HUWE1 docks onto a helical scaffold within HAPSTR1 (Fig. 3B; residues 23 to 134). HAPSTR1 binding surface centers on residues A96, T98, and K102 along the same α-helical face (Fig. 3B). Deletion of A96 is thus expected to disrupt the binding interface, reorienting key interface residues of HAPSTR1 and weakening helix-helix complementarity. To test this, we analyzed coprecipitates of GFP-HAPSTR1 versus GFP-ΔA96 by mass spectrometry (MS). As expected, HUWE1 was identified as the top interactor for wild-type HAPSTR1, along with USP7 and BAG6, and binding to HUWE1 was abrogated upon A96 deletion from HAPSTR1 (Fig. 3D, fig. S3D, and data S2). Although the impact of HUWE1 mutants was not assessed in this context, and potential for indirect binding cannot be unequivocally ruled out, ubiquitin signaling suppression had no effect on the association of HUWE1 with HAPSTR1 (Fig. 3D and fig. S3E), indicating that complex formation between them is ubiquitination independent. Together with the data presented in Figs. 1 and 2, the above considerations advance the hypothesis that HAPSTR1 and HUWE1 work together to promote cellular proliferation.

**Fig. 3.**
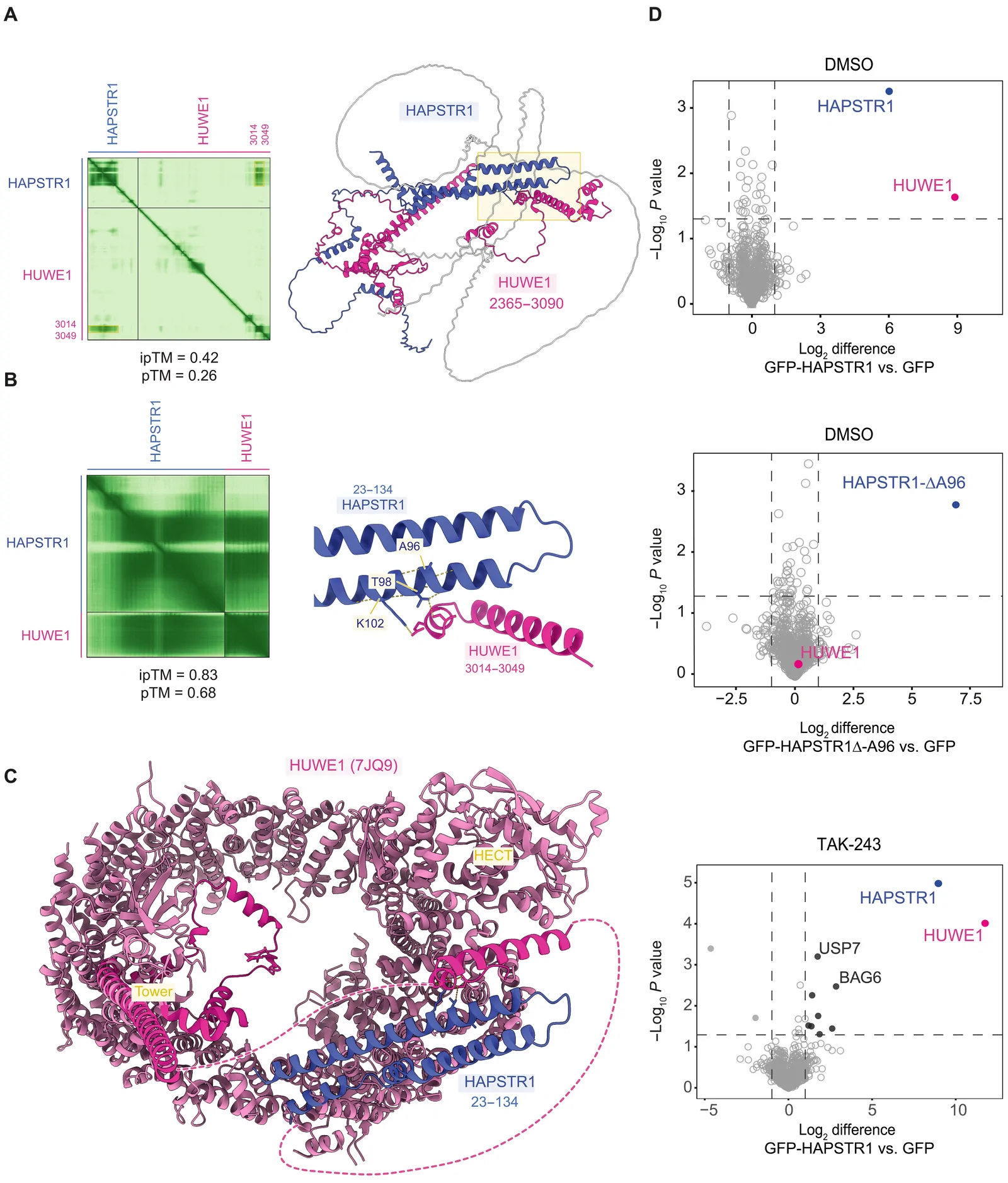
Structural modeling of the HAPSTR1/HUWE1 complex. (**A** to **C**) Modeling the interaction interface between HAPSTR1 and HUWE1. AlphaFold3 modeling of (A) HUWE1 amino acids 2365 to 3090 interacting with full-length HAPSTR1 and (B) HUWE1 amino acids 3014 to 3049 interacting with HAPSTR1 amino acids 23 to 134. (C) Docking of the AlphaFold model onto full-length HUWE1 (PDB 7JQ9). (**D**) Protein-protein interaction profiling of GFP-HAPSTR1. GFP-Trap precipitates from RPE1-p53^−/−^ cells induced to express GFP, GFP-HAPSTR1, or GFP-ΔA96 and cultured in the absence or presence of TAK-243 were analyzed by MS. Volcano plots of proteins identified over GFP (control) are shown. Blue: HAPSTR1, magenta: HUWE1, and black: other. Significance determined by two-sided *t* testing, *P* < 0.05 and *S*_0_ = 0.1 based on *n* = 3 independent experiments. See also fig. S3 and data S2.

### HAPSTR1 recruits HUWE1 to the chromatin to regulate chromatin proteostasis

HAPSTR1 has recently been described as a rheostat for cellular stress pathways (*20*), and HAPSTR1-dependent nuclear localization of HUWE1 was shown to promote clearance of damaged, aggregated, or partially ubiquitinated proteins (*21*, *24*). In seeking to understand the mechanistic basis of synthetic lethality observed between HAPSTR1 loss and ubiquitination stress via TAK-243, we considered a possible connection through chromatin-associated proteostasis. To explore this, we generated clonal HAPSTR1 KO RPE1-p53^−/−^ cell lines (fig. S4A) and used high-sucrose separation methodology, followed by MS to test whether loss of HAPSTR1 affects protein composition at the chromatin (fig. S4, B and C, and data S3). This revealed accumulation of mitotic regulators (e.g., CDK1, KIF22, and KIF18A) and ubiquitin machinery components, including CUL2 and TRIM26, at the chromatin of HAPSTR1-KO cells relative to control (Fig. 4A and fig. S4C). By contrast, HUWE1 was depleted from the chromatin fractions of HAPSTR1-deficient cells (Fig. 4A and fig. S4C). Immunoblot analysis confirmed that, while total HUWE1 levels remained unchanged, its association with the chromatin diminished in the absence of HAPSTR1, irrespective of TAK-243 treatment (Fig. 4, B and C). Moreover, HAPSTR1 itself was present in the chromatin fraction (Fig. 4D). Because HAPSTR1 contains a nuclear localization sequence (NLS), these observations provide a rationale for the recruitment of HUWE1, which lacks its own NLS.

**Fig. 4.**
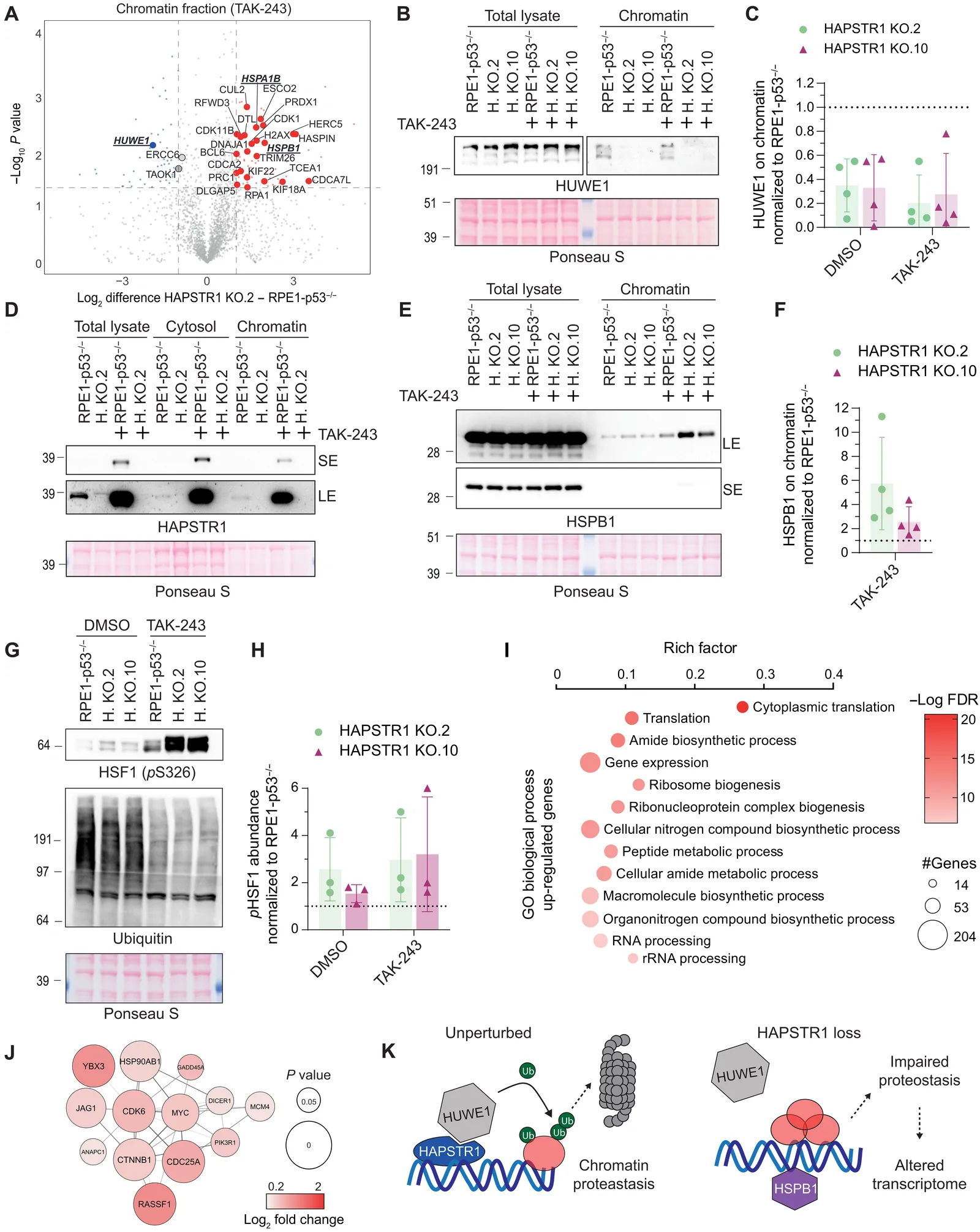
Depletion of HUWE1 from chromatin, disturbed chromatin proteostasis, and transcriptional rewiring in HAPSTR1-deficient cells. (**A** to **F**) Impact of HAPSTR1 ablation on chromatin-associated proteome. (A) Volcano plot of chromatin-bound proteins enriched (red) or depleted (blue) in HAPSTR1-KO.2 cells compared to RPE1-p53^−/−^ cells, treated with TAK-243 (right). FDR set to 1%; data representative of *n* = 4 independent experiments. Representative immunoblots of (B) HUWE1, (D) HAPSTR1, and (E) HSPB1 protein levels in total lysates (TL) and chromatin fractions from RPE1-p53^−/−^, HAPSTR1-KO.2, and HAPSTR1-KO.10 cells, treated with TAK-243 where indicated (+); long exposure (LE), short exposure (SE), and Ponceau S: loading control. Quantification of chromatin-associated (C) HUWE1 and (F) HSPB1 band intensities (mean ± SD) normalized to RPE1-p53^−/−^ from *n* = 4 independent experiments. See also fig. S4 and data S3. (**G** and **H**) Effect of HAPSTR1 ablation on heat shock response. (G) Representative immunoblots against phospho-HSF1 (pHSF1) in RPE1-p53^−/−^, HAPSTR1-KO.2, and HAPSTR1-KO.10 cells, treated with TAK-243 (1 μM for 17 hours) where indicated (+); Ponceau S: loading control. (H) Graph reports pHSF1 band intensities (mean ± SD), normalized to RPE1-p53^−/−^ from *n* = 3 independent experiments. (**I**) Impact of HAPSTR1 ablation on the transcriptome. Top 20 enriched GO biological processes using STRING analysis (redundancy cutoff = 1) of overlapping up-regulated (red) genes (*P* < 0.05) in HAPSTR1-KO.2 and HAPSTR1-KO.10 cells relative to RPE1-p53^−/−^ identified by RNA sequencing (RNA-seq). Bubble size: number of genes involved in each process and color intensity: –log FDR value. See also data S4. (**J**) Cluster of CDK6-associated genes up-regulated in HAPSTR1-KO.2 and HAPSTR1-KO.10 cells relative to RPE1-p53^−/−^ from (I). Node size: *P* value; color: log_2_ fold change. (**K**) Model of HAPSTR1 function in chromatin proteostasis. HAPSTR1 promotes association of HUWE1 with the chromatin and thus supports ubiquitination-dependent protein clearance. Cells lacking HAPSTR1 up-regulate cell cycle proteins to sustain cellular proliferation.

We further noted that HAPSTR1 loss evokes up-regulation of heat shock chaperone proteins HSPB1 and HSPA1B at the chromatin, specifically under conditions of compromised ubiquitin signaling (Fig. 4, A, E, and F, and fig. S4C). These chaperones accumulate in the nucleus in the presence of genotoxic and proteotoxic perturbations, where they safeguard protein quality control and facilitate cellular adaptations to stress (*25*–*27*). We therefore hypothesized that accumulation of chromatin-bound HSPB1 in HAPSTR1-KO cells, without changes in total HSPB1 levels, reflects a compensatory response to impaired HUWE1-mediated chromatin proteostasis. Supporting this, we observed elevated levels of phosphorylated HSF1 (pHSF1) in HAPSTR1-KO cells (Fig. 4, G and H), a master transcriptional regulator of the heat shock response, which becomes phosphorylated upon proteotoxic stress (*28*). We picked up loss of HSF1 as a sensitizing factor to TAK-243 administration in our chemical-genetic screen (fig. S1E), alongside loss of HAPSTR1 and HUWE1, indicating that all three proteins are required for proliferating cells to cope with the ubiquitin signaling blockade.

Given the accumulation of stress-related chaperones at the chromatin observed under conditions of supressed ubiquitination, we hypothesized that disrupted chromatin proteostasis—and the resulting stress incurred in HAPSTR1-deficient cells—could induce broader adaptations through altered gene expression. In line with previous reports (*20*, *21*), we observed global transcriptional changes spanning diverse pathways as a function of HAPSTR1 loss. Specifically, we identified 414 up-regulated protein-coding genes to be significantly altered in two independent HAPSTR1-KO cell lines, relative to RPE1-p53^−/−^ (Fig. 4I and data S4). Gene Ontology analysis revealed up-regulation of cell cycle regulators and genes involved in translation and RNA processing in response to HAPSTR1 ablation (Fig. 4, I and J), suggesting that loss of HAPSTR1 remodels gene expression to boost cellular proliferation and survival. Together, these observations point to a positive role for HAPSTR1 in ubiquitin-dependent proteostasis at the chromatin (Fig. 4K) and provide a rationale for the synthetic lethality of HAPSTR1 ablation with ubiquitin signaling blockade.

### HAPSTR1 dynamics throughout the cell cycle 

Given the notable effects of HAPSTR1 ablation on cellular proliferation (Fig. 2, A to C) and the established connection between cell cycle progression and chromatin proteostasis (*29*), we wondered whether HAPSTR1 expression exhibits cell cycle dynamics. To explore this, we visualized ectopically expressed fluorescently tagged HAPSTR1 by confocal time-lapse microscopy. Consistent with its C-terminal NLS (fig. S3B), GFP-HAPSTR1 and GFP-ΔA96 localized in the nuclei of interphase cells (fig. S5A). Higher levels of both N- and C-terminally tagged HAPSTR1 during G_2_ were observed, declining sharply following mitosis (Fig. 5, A and B, and fig. S5, B and C). In line with previous studies demonstrating HUWE1-mediated ubiquitination and degradation of HAPSTR1 (*20*, *21*), turnover of HAPSTR1 in G_1_ phase was strongly diminished upon treatment with TAK-243 or knockdown of HUWE1 (Fig. 5B). Accordingly, treatment with TAK-243 or the proteasome inhibitor MG132 stabilized endogenous HAPSTR1 (Fig. 5C), and ubiquitination of HAPSTR1 was strongly enhanced upon MG132 treatment (Fig. 5D), indicating UPS-dependent turnover.

**Fig. 5.**
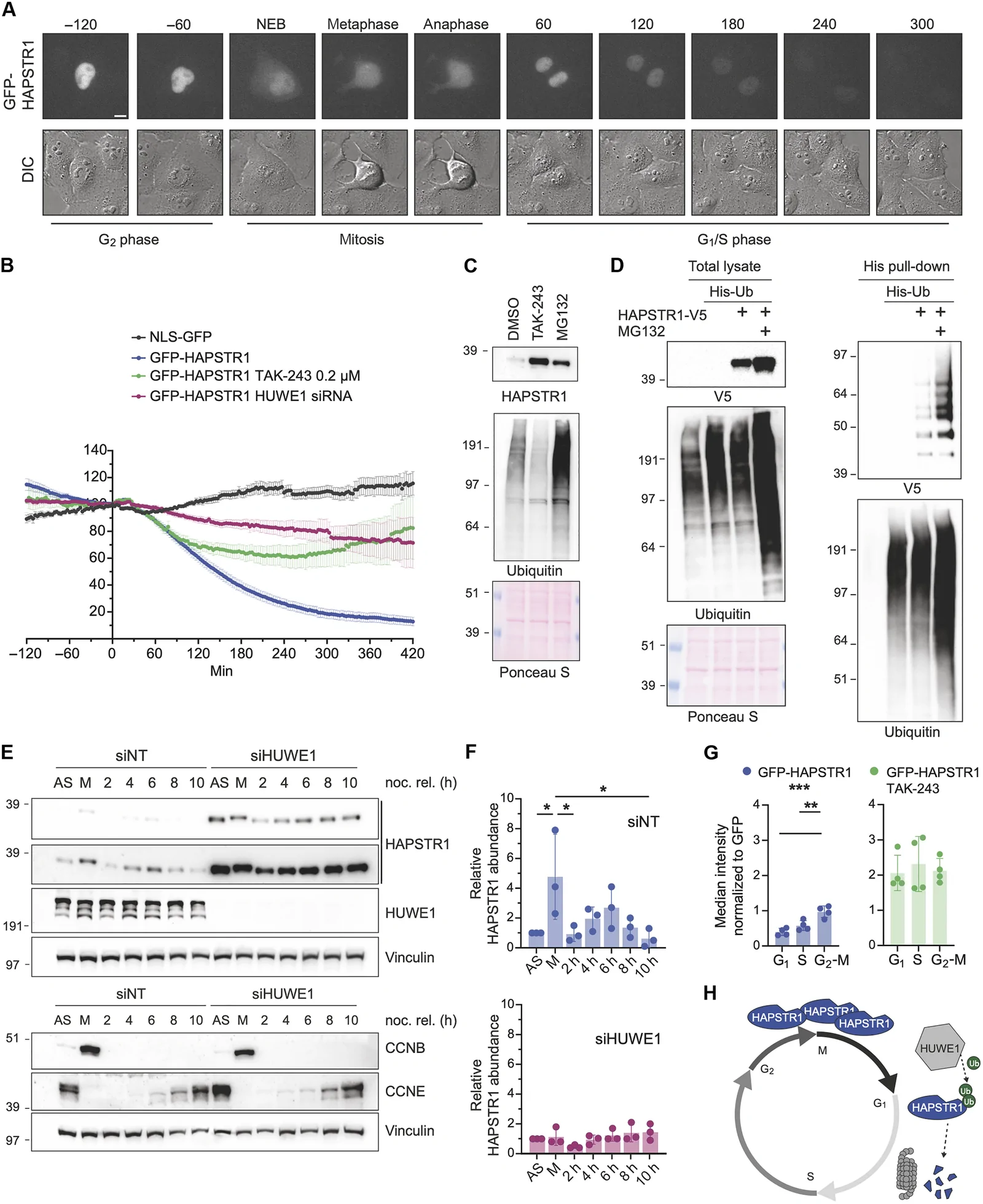
HAPSTR1 level dynamics throughout the cell cycle. (**A**) Representative confocal fluorescence images of dividing U2OS cells ectopically expressing GFP-HAPSTR1 (3 min per frame). Scale bar, 5 μm. (**B**) Average GFP-HAPSTR1 and nuclear localization signal (NLS)–GFP (control) fluorescence intensities (mean ± SEM) plotted over time (in minutes) in cells treated with dimethyl sulfoxide (DMSO; blue), TAK-243 (0.2 μM; green), or HUWE1 small interfering RNA (siRNA) (10 mM for 48 hours; purple); NEB (nuclear envelope breakdown; *t* = 0). DMSO: *n* = 3 independent experiments; *N* = 44 cells. TAK-243: *n* = 5 independent experiment; *N* = 41 cells. HUWE1 siRNA (siHUWE1): *n* = 4 independent experiments; *N* = 23 cells. NLS-GFP: *n* = 3 independent experiments; *N* = 37 cells. See also fig. S5. (**C** and **D**) Effects of UPS inhibition on HAPSTR1 stability and ubiquitination status. Representative immunoblots (*n* = 3 independent experiments) of (C) RPE1-p53^−/−^ cells treated with TAK-243 (1 μM for 3 hours) or proteasome inhibitor MG132 (10 μM for 3 hours) or (D) TL and nickel–nitrilotriacetic acid (Ni-NTA) precipitates from RPE1-p53^−/−^ cells expressing His-Ub and V5-HAPSTR1, treated with MG132 (10 μM for 6 hours). Ponceau S: loading control. (**E** to **G**) Cell cycle–dependent dynamics of HAPSTR1. RPE1-p53^−/−^ cells, transfected with nontargeting (siNT) or siHUWE1, were synchronized (nocodazole) at mitosis (M) and released (0 to 10 hours). (E) Representative immunoblot analyses of endogenous HAPSTR1, vinculin (control), CCNB, and CCNE (synchronization controls); AS (asynchronous). h, hours. (F) Quantification of HAPSTR1 abundance relative to AS [*n* = 3 independent experiments; mean ± SD, one-way analysis of variance (ANOVA) with Tukey’s multiple-comparison test, *P < 0.05]. (G) Flow cytometry analysis of RPE1-p53^−/−^ cells stably expressing dox-inducible GFP-HAPSTR1 or GFP (control), treated with DMSO (blue) or TAK-243 (17 hours, 0.5 μM; green), and stained with 4′,6-diamidino-2-phenylindole (DAPI; DNA content) to distinguish G_1_, S, and G_2_-M cell populations (*n* = 4 independent experiments; mean ± SD, two-way ANOVA with Tukey’s multiple-comparison test, **P < 0.01 and ***P < 0.001). See also fig. S6. (**H**) Model of HAPSTR1 dynamics across the cell cycle. HAPSTR1 is highly expressed during G_2_ and M, followed by HUWE1-dependent ubiquitination and degradation in G_1_.

To further investigate endogenous HAPSTR1 dynamics across the cell cycle, we synchronized cells at prometaphase using nocodazole and analyzed protein expression by immunoblotting as a function of time following washout. Consistently, cells maintained higher levels of HAPSTR1 during mitosis, coinciding with high expression of the mitotic marker CCNB, and down-regulated HAPSTR1 in G_1_ (Fig. 5, E and F, and fig. S6, A to D). Depletion of HUWE1 markedly stabilized HAPSTR1 across the measurement window, eliminating degradation in G_1_ (Fig. 5, E and F). Lastly, monitoring protein abundance across the entire cell cycle by flow cytometry revealed higher levels of GFP-HAPSTR1 in the G_2_-M cell population compared to those in G_1_ and S (Fig. 5G and fig. S6, E and F). Here, inhibition of ubiquitin signaling by TAK-243 stabilized GFP-HAPSTR1 and thus abolished its cycling (Fig. 5G and fig. S6F). These results demonstrate dynamic control of HAPSTR1 protein expression across the cell cycle in a UPS-dependent manner, where high expression during G_2_ and M gives way to HUWE1-dependent ubiquitination and degradation in G_1_ (Fig. 5H). Such regulation of HAPSTR1 may therefore provide a feedback mechanism enabling temporal control of HUWE1 recruitment to the chromatin.

### Rewired ubiquitinome reveals early activation of the CDK6 pathway upon HAPSTR1 loss

Building on the positive role of HAPSTR1 in chromatin proteostasis and its connection to the ubiquitin ligase HUWE1, we explored the impact of HAPSTR1 loss on the global cellular ubiquitination landscape. To map the ubiquitinome of control RPE1-p53^−/−^ cells versus those lacking HAPSTR1, we used the UbiSite technique, allowing purification and analysis of endogenous ubiquitin substrates from cells treated with MG132 to inhibit proteasomal degradation of ubiquitin conjugates (Fig. 6A) (*30*). The ubiquitinome changed substantially in the absence of HAPSTR1, revealing divergent ubiquitination of 7942 sites in 1801 proteins (data S5), with notable presence of cell cycle regulators (Fig. 6B). Functional enrichment analysis further highlighted increased ubiquitination of transcriptional pathway regulators in response to HAPSTR1 ablation (Fig. 6C).

**Fig. 6.**
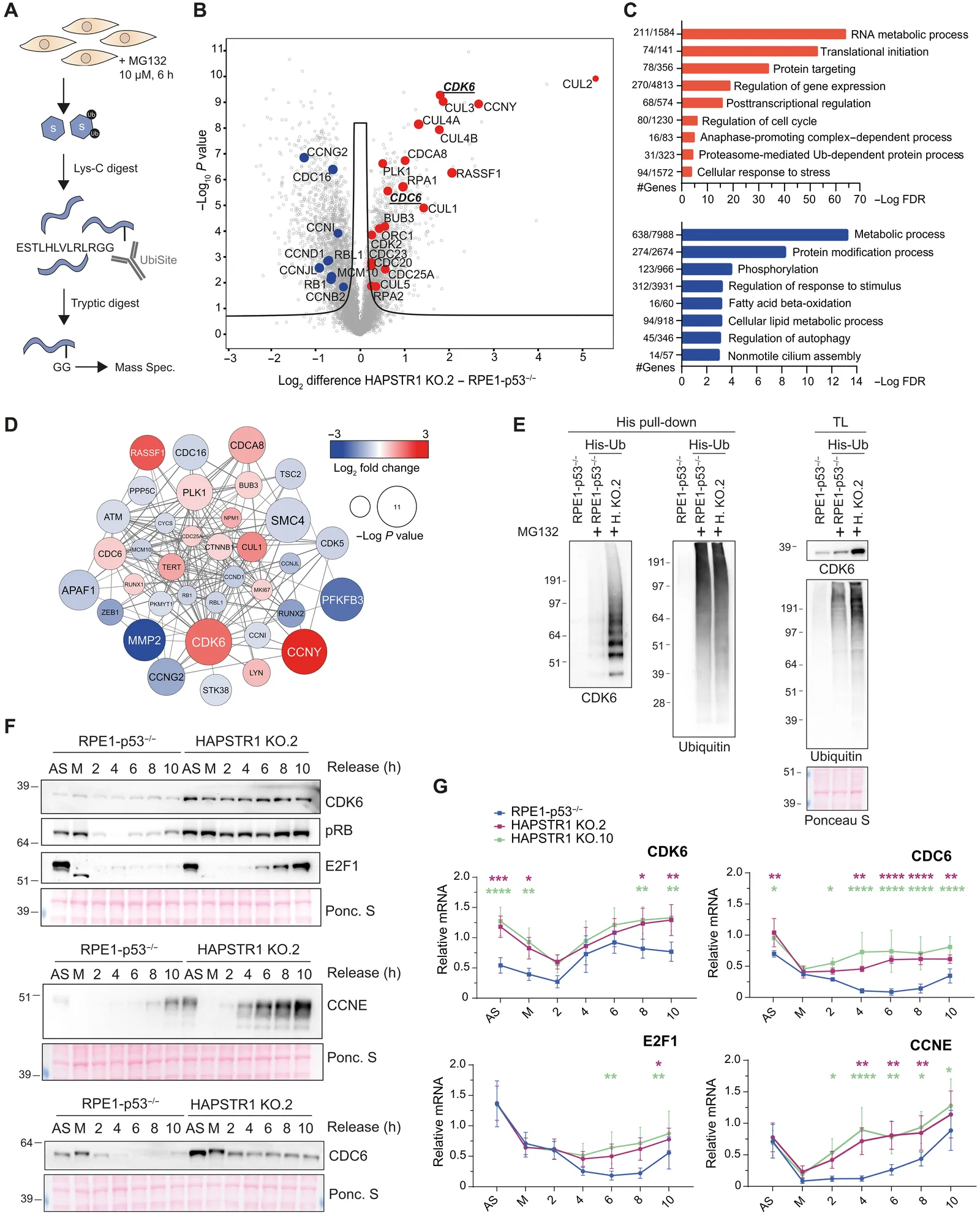
HAPSTR1 loss triggers hyperactivation of the CDK6 pathway. (**A** to **D**) Impact of HAPSTR1 ablation on the ubiquitinome. (A) Schematic of UbiSite technique using antibody-based recognition of ubiquitinated protein remnants following Lys-C digestion. Ubiquitinated proteins, isolated under denaturing conditions from cells treated with MG132 (10 μM for 6 hours), were analyzed by MS. (B) Volcano plot and (C) GO biological process annotation of ubiquitinated proteins found to be enriched (red) or depleted (blue) in HAPSTR1-KO.2 cells compared to control RPE1-p53^−/−^ cells (representative of *n* = 5 independent experiments; statistical analysis performed using two-sided student’s *t* test with permutation-based FDR of 0.05 and *S*_0_ = 0.1). (D) CDK6 cluster of proteins significantly altered in the ubiquitome of HAPSTR1-KO.2 cells identified using STRING network analysis (confidence cutoff = 0.4). Node size: –log *P* value; color: log_2_ fold change; edge width: protein-protein interaction score. See also data S5. (**E**) Effects of HAPSTR1 loss and UPS inhibition on CDK6 ubiquitination. Representative immunoblot analyses (*n* = 3 independent experiments) of endogenous CDK6 and Ub in TL and Ni-NTA precipitates from RPE1-p53^−/−^ and HAPSTR1-KO.2 cells expressing His-Ub and treated with proteasome inhibitor MG132 (10 μM for 6 hours) where indicated (+); Ponceau S: loading control. See also fig. S7. (**F** and **G**) Effects of HAPSTR1 ablation on CDK6 pathway activation. (F) Representative immunoblot analyses (*n* = 3 independent experiments) against the indicated proteins in lysates from RPE1-p53^−/−^ and HAPSTR1-KO.2 cells, AS or synchronized in M and released (2 to 10 hours); Ponceau S: loading control. (G) Reverse transcription–qPCR analysis of RPE1-p53^−/−^ (blue), HAPSTR1-KO.2 (magenta), and HAPSTR1-KO.10 (green) cells treated as in (F). Graphs report mRNA levels normalized to housekeeping genes (*n* = 4 independent experiments; mean ± SD, two-way ANOVA with Tukey’s multiple-comparison test, *P < 0.05 **P < 0.01, ***P < 0.001, and ****P < 0.0001). See also figs. S8 and S9.

In the ubiquitinome of HAPSTR1-KO cells, CDK6 was found to exhibit a striking 3.5-fold increase in ubiquitination (Fig. 6B) across all seven ubiquitin acceptor sites identified in CDK6 (fig. S7A). Furthermore, STRING analysis highlighted a strong interconnected cluster of interacting partners of CDK6 (*31*, *32*), all displaying altered ubiquitination in response to HAPSTR1 loss (Fig. 6D). To validate UbiSite findings, we performed His-ubiquitin (His-Ub) precipitation, confirming elevated CDK6 ubiquitination in HAPSTR1-KO cells (Fig. 6E and fig. S7, B and C). CDK6 ubiquitination was also detectable under unperturbed conditions and was further enhanced under proteasome blockade in RPE1-p53^−/−^ cells, indicating that CDK6 is subject to ubiquitin-proteasomal regulation, even in the presence of HAPSTR1 (fig. S7D).

We next examined the effect of HAPSTR1 ablation on the CDK6 signaling pathway. The established CDK6 pathway involves CDK6/CCND-mediated phosphorylation of Rb, which releases E2F1 from repression by Rb, causing its activation. Activated E2F1, in turn, initiates transcription of S phase entry drivers CCNE and E2F1 (*33*). Using nocodazole synchronization, verified by immunostaining of phosphorylated histone H3, MPM2, and CCNB (fig. S8A), we monitored expression levels of CDK6 and its pathway effectors for up to 10 hours postrelease. CDK6 protein abundance was markedly elevated in cells lacking HAPSTR1, and this was accompanied by increased phosphorylation of Rb (Fig. 6F and fig. S8, B and C). Besides CDK6, CDC6 also exhibited elevated ubiquitination in HAPSTR1-KO cells, relative to RPE1-p53^−/−^ (Fig. 6D). This was similarly accompanied by an increase in overall CDC6 protein abundance (Fig. 6F and fig. S8, B and C). In contrast to CDK6 and CDC6, no up-regulation of CDK4 (a close relative of CDK6) or CCND was observed under the same conditions (fig. S8, C and D), demonstrating specificity. Consistent with heightened CDK6 activity, HAPSTR1 ablation gave rise to higher levels of downstream effectors CCNE and E2F1 from 4 to 6 hours postmitotic release (Fig. 6F and fig. S8, B and C), indicating premature pathway activation.

In line with the above observations, CDK6 (but not CDK4) was transcriptionally up-regulated in our global gene expression analysis in cells lacking HAPSTR1 (Fig. 4J), which was confirmed by quantitative polymerase chain reaction (qPCR) analysis (fig. S9A). Moreover, transcription of CDK6 associated proteins, RASSF1 and CDC25A, both of which are known to play active roles at the G_1_-S transition (*31*, *32*), was similarly enhanced (Fig. 4J), implying a broader influence of HAPSTR1 on the CDK6 pathway gene expression. Synchronization experiments showed that cells lacking HAPSTR1 up-regulate CDK6 and CDC6 transcripts throughout G_1_, as well as in the asynchronous population (Fig. 6G). By contrast, early transcriptional increases of downstream effectors E2F1 and CCNE remain restricted to the S phase transition (Fig. 6G), suggesting that the latter may result from early activation of the CDK6 pathway rather than constitute a direct effect of HAPSTR1 loss. As expected, mRNA levels of CDK4 and CCND remained largely unaffected in the absence of HAPSTR1 (fig. S9B), reflecting specificity. Collectively, these observations further the notion that transcriptional reprogramming and ubiquitination-driven adaptations impinge on common cell cycle regulators to sustain cellular proliferation in HAPSTR1-deficient cells.

### Loss of HAPSTR1 accelerates S phase entry and disrupts cell cycle integrity

Given the cell cycle–dependent expression of HAPSTR1 and its effects on CDK6 pathway activation, we hypothesized that progression through the cell cycle would be negatively affected by ablation of HAPSTR1, particularly under the added pressures of ubiquitin signaling blockade. To test this, we treated asynchronous parental RPE1-p53^−/−^ and HAPSTR1-KO cells with the thymidine analog 5-ethynyl-2′-deoxyuridine (EdU; label for cells undergoing DNA replication) and subjected to fluorescence-activated cell sorting (FACS) analysis to determine the proportion of cells in each cell cycle phase. In cells devoid of HAPSTR1, the G_1_ fraction was slightly diminished, accompanied by an increase in the G_2_-M content as compared to RPE1-p53^−/−^ (Fig. 7, A and B, and fig. S10, A and B). Notably, treatment with TAK-243 greatly exacerbated the observed differences across the cell cycle, including prominent accumulation of cells at the start of S phase, and concomitant decrease in the G_1_ population (Fig. 7, A and C, and fig. S10, C to G). These changes became evident as early as 3 hours of TAK-243 treatment in HAPSTR1-KO cells (fig. S10, E and F). Moreover, cells lacking HAPSTR1 displayed impaired EdU incorporation, resulting in loss of EdU-positive cells (S phase) and an increase in incorporation defective cells that feature aberrant replication dynamics (Fig. 7, A and C, and fig. S10, A to G). In line with this, flow-cytometry analysis following nocodazole synchronization revealed that HAPSTR1-KO cells exhibit an accelerated transition from G_1_ to S phase and even in the absence of ubiquitin stress a modest decrease in G_1_ (Fig. 7D).

**Fig. 7.**
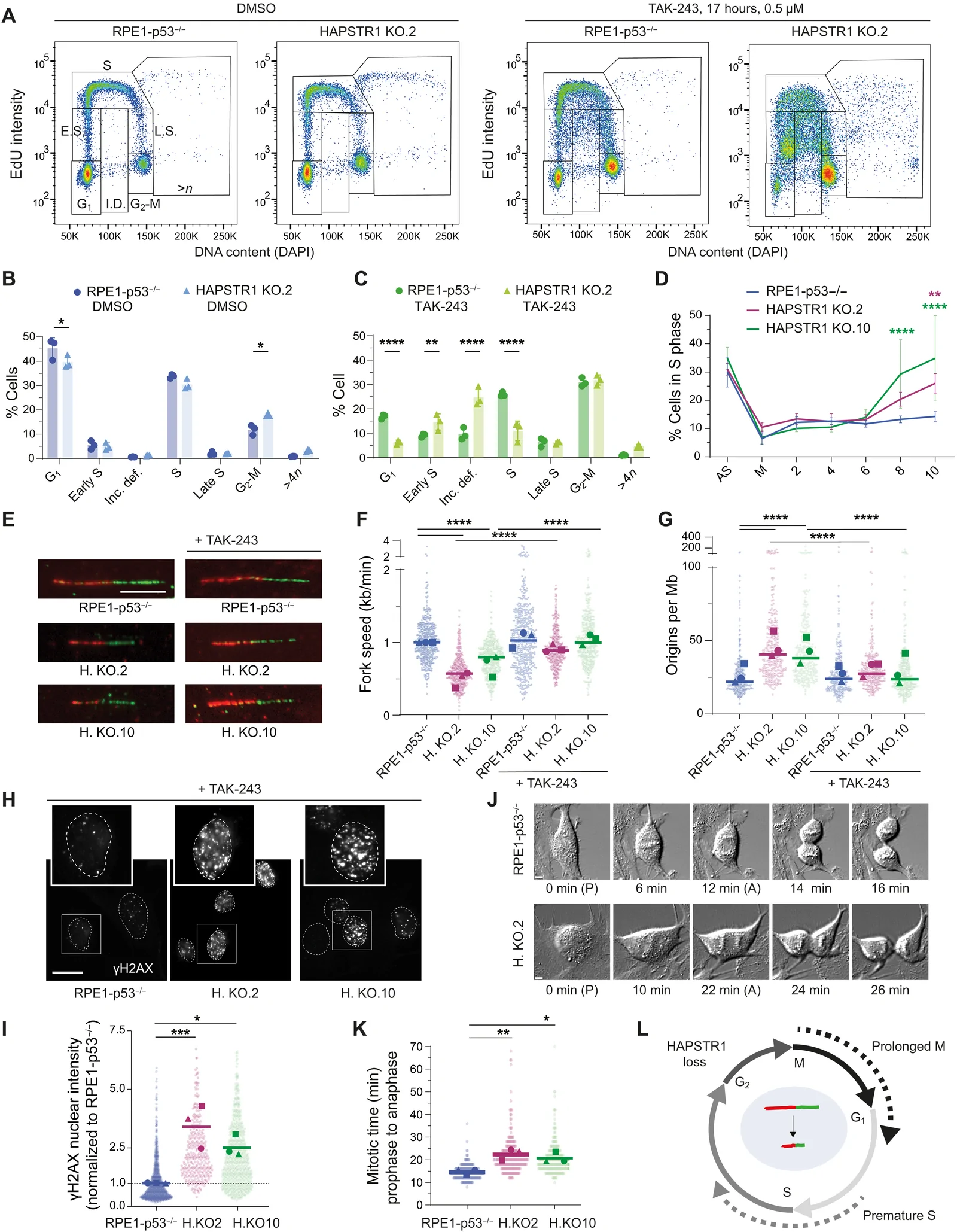
HAPSTR1 loss incurs genomic instability and cell cycle collapse. (**A**) Flow cytometry analyses of DAPI (DNA content) and EdU (DNA replication) in RPE1-p53^−/−^ versus HAPSTR1-KO.2 cells. Graphs report the percentage of cells per cell cycle phase (G_1_, S, G_2_, and M; E.S.: early S phase, L.S.: late S phase, and I.D. and Inc. def.: incorporation defects) are plotted for (**B**) DMSO (blue) and (**C**) TAK-243 (green) (*n* = 3 independent experiments; mean ± SD). (**D**) Percentages (%) of AS, M, and postmitotic RPE1-p53^−/−^ (blue) versus HAPSTR1-KO.2 (magenta) and HAPSTR1-KO.10 (green) cells (*n* = 4 independent experiments; mean ± SD). Two-way ANOVA with Tukey’s multiple-comparison test; *P < 0.05, **P < 0.01, and ****P < 0.0001. See also fig. S10. (**E**) Representative images of DNA fibers from cells treated with TAK-243, for 17 hours (0.5 μM), where indicated. Quantification of (**F**) replication fork speed (*n* = 450 fibers per condition) and (**G**) interorigin distance (IOD; *n* = 300 fibers per condition) in RPE1-p53^−/−^ and HAPSTR1-KO cells depicting individual fibers (transparent datapoints) and mean values per experiment plotted as solid datapoints. (**H**) Representative images of DNA damage marker γH2AX (white) in RPE1-p53^−/−^ and HAPSTR1-KO cells treated with TAK-243 for 17 hours (0.5 μM). Cell nuclei are demarcated with dashed lines. Scale bar, 10 μm. (**I**) Quantification of γH2AX fluorescence intensity. Graph depicts individual cells (*n* > 300 per condition; transparent datapoints) and mean values (solid datapoints) per experiment (*n* = 3 independent experiments; one-way ANOVA with Tukey’s multiple-comparison test, *P < 0.05 and ***P < 0.001). (**J**) Representative differential interference contrast (DIC) images from dividing RPE1-p53^−/−^ and HAPSTR1-KO.2 cells (2 min per frame). Scale bars, 5 μm. (**K**) Graph reports duration (in minutes) from prophase (P) to anaphase (A) of individual cells (transparent datapoints) and mean values (solid datapoints) per experiment (*n* = 3 independent experiments; one-way ANOVA with Tukey’s multiple-comparison test, *P < 0.05 and **P < 0.01). See also fig. S11. (**L**) HAPSTR1 deficiency leads to premature S phase entry and lagging mitotic progression.

To investigate whether premature S phase entry observed above hampers DNA replication, we used DNA fiber assays (*34*). Quantification of DNA track length showed decreased replication fork speed in HAPSTR1-KO cells (Fig. 7, E and F), indicating replication stress.

Treatment with TAK-243 restored replication fork speed in HAPSTR1-KO cells to levels comparable to those in RPE1-p53^−/−^ cells (Fig. 7, E and F). Given that asynchronous HAPSTR1-KO cells specifically accumulated at the start of S phase upon TAK-243 treatment (Fig. 7, A to C), we wondered how these cells could compensate for slowed replication progress. Deducing the number of origins per mega–base pairs (Mbp) from the interorigin distance (IOD) showed an increase in origin firing in HAPSTR1-KO cells, which correlated with slower forks (Fig. 7G). Accordingly, treatment with TAK-243 restored the number of origins fired in HAPSTR1-KO cells, as seen for the replication fork speed (Fig. 7G). We hypothesized that proceeding with replication under conditions of deregulated proteostasis at the chromatin would come at the expense of DNA damage. In support of this, HAPSTR1-KO cells exhibited a heightened γH2AX response relative to RPE1-p53^−/−^ cells (Fig. 7, H and I). Moreover, HAPSTR1-deficient cells expressed elevated protein (but not transcript) levels of the CDKI p27^Kip1^ and lower levels of p21^Cip1^ (fig. S11, A and B) (*35*, *36*), implying activation of the G_1_-S checkpoint to delay cell cycle progression. Last, we observed lagging mitotic progression in HAPSTR1-deficient cells as compared to RPE1-p53^−/−^ (Fig. 7, J and K), signaling a possible compensatory response to aberrant S phase entry. Collectively, our data indicate that HAPSTR1 contributes to cell cycle integrity (Fig. 7L).

Together, our findings support a model in which HAPSTR1 plays a central role in the maintenance of chromatin proteostasis through recruitment of HUWE1 to the chromatin. HUWE1, in turn, regulates HAPSTR1 stability, targeting HAPSTR1 for degradation in G_1_, which sets up a feedback mechanism to temporally control HUWE1-chromatin interactions (Fig. 8A). Loss of HAPSTR1 disrupts clearance of chromatin-associated proteins and triggers widespread deregulation of the transcriptional landscape and cell cycle progression. In response, HAPSTR1-deficient cells up-regulate factors such as CDK6 and CDC6 to accelerate S phase entry and sustain origin firing in the background of replication stress. Here, suppressed UPS function renders cells unable to exercise dynamic control over their proteomes or effectively activate cell cycle checkpoints (*13*, *37*, *38*). HAPSTR1-deficient cells may develop compensatory mechanism due to deficient ubiquitin signaling to partially overcome loss of HAPSTR1. Combinatorial loss of HAPSTR1 and ubiquitin signaling thus synergize to compromise cell cycle integrity, leading to aberrant proliferation and cell death (Fig. 8B).

**Fig. 8.**
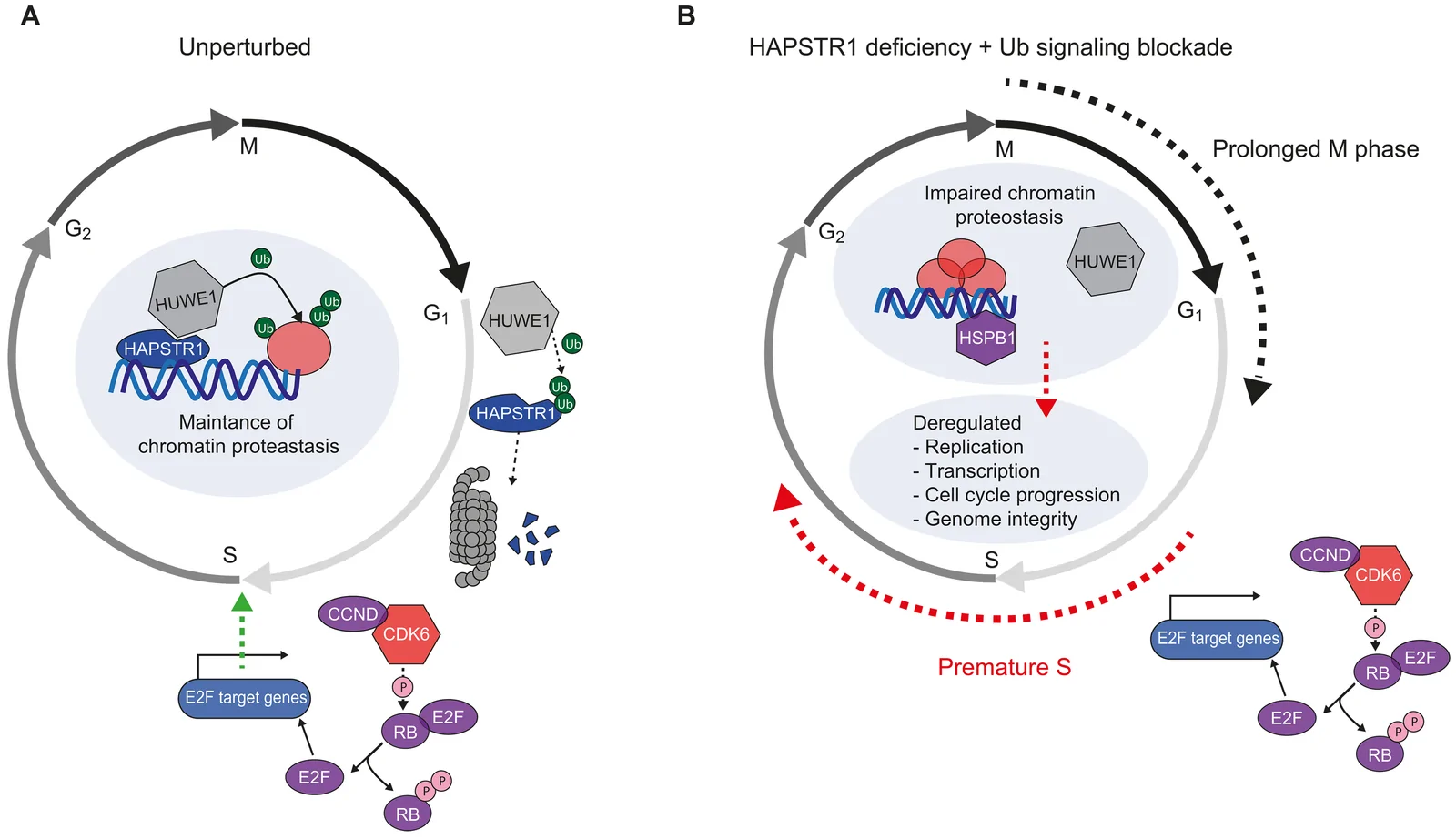
Model for the role of the HAPSTR1-HUWE1 axis in the maintenance of chromatin proteostasis and cell cycle integrity. (**A** and **B**) Proposed model. (A) HAPSTR1 maintains dynamic chromatin proteostasis through recruitment of HUWE1 to the chromatin, thereby positively contributing to cell cycle progression and fidelity. HUWE1, in turn, down-regulates HAPSTR1 in G_1_. (B) In the absence of HAPSTR1, chromatin proteostasis is deregulated, leading to disturbances in cell cycle processes. HAPSTR1-KO cells experience global transcriptional alterations, aimed at sustaining cell proliferation in the face of disturbed chromatin proteostasis. Under conditions of suppressed ubiquitin signaling, proteotoxic stress at the chromatin of HAPSTR1-KO cells is further exacerbated, resulting in the activation of HSF1 and recruitment of chaperones. Because cells depend on the ubiquitin system to exercise control over cell cycle progression and proteostasis, HAPSTR1 loss combined with TAK-243 treatment induces replication defects and premature S phase entry downstream of early CDK6 pathway activation, leading to cell cycle collapse and the accumulation of DNA damage.

## DISCUSSION

In this study, we used a chemical-genetic CRISPR KO screen to explore the interplay between ubiquitin signaling and protein networks governing cellular proliferation. Harnessing the ubiquitin E1 inhibitor TAK-243 as a powerful tool to suppress the ubiquitination cascade, we now present a comprehensive characterization of ubiquitin signaling components that are essential for cell proliferation. As pressure on UPS function constitutes a common feature of prominent human pathologies, including cancer (*4*–*6*) and neurodegeneration, understanding cellular responses to ubiquitin signaling blockade is of profound significance. Here, we uncover a subset of tumor-suppressor genes whose impact on proliferation is magnified in the context of hampered ubiquitin signaling and reveal key molecular players critical for proliferating cells to cope with UPS disruption.

Among the top genes whose ablation sensitized cells toward ubiquitin blockade, we identified HAPSTR1—a protein adaptor and substrate of the E3 ubiquitin ligase HUWE1 (*20*, *21*). We carried out structural modeling of the HAPSTR1/HUWE1 complex and validated the model by point mutation of HAPSTR1. We link this interaction to cell cycle control by demonstrating that genomic HAPSTR1 point mutations abrogating HUWE1 binding behave as full KOs in the context of cellular proliferation. This extends previous observations of the interaction of HAPSTR1 and HUWE1 that lack structural modeling of this interaction (*20*, *21*). On the basis of its recent identification as synthetic lethal with poly(adenosine 5′-diphosphate–ribose) polymerase 1/2 gene disruption, ATR inhibition, and telomerase inhibition (*39*–*41*), HAPSTR1 has been described as a central regulator of stress response pathways (*20*). Our work extends this emerging paradigm by implicating HAPSTR1 in chromatin proteostasis through its control of HUWE1 recruitment to the chromatin. This extends the literature where it was previously shown that HAPSTR1 is required for nuclear location of HUWE1 (*21*, *24*). We further show that HAPSTR1 is regulated by the UPS at different phases of the cell cycle in a HUWE1 dependent manner, suggesting a feedback mechanism to control dynamic association of HUWE1 with the chromatin. While loss of HAPSTR1 reduces HUWE1 levels at the chromatin, temporal triggers for ubiquitination and clearance of HAPSTR1 remain to be clarified. Considering that HAPSTR1 accumulates following genome duplication (during G_2_ phase and in mitosis) and is rapidly degraded postmitosis (onset of G_1_ phase), it is attractive to speculate that the HAPSTR1/HUWE1 axis oversees clearance of chromatin-associated proteins in the lead up to mitosis, as chromatin condenses to facilitate chromosome alignment and segregation. Following cell division, chromatin decondenses in early G, allowing transcriptional activity to resume. Later in the cell cycle, as HAPSTR1 expression recovers, enhanced protein clearance from chromatin can once again be called upon to prepare cells for replication. In this setting, it can be envisioned that, once chromatin affiliated substrates of HUWE1 are consumed, HUWE1 becomes available to ubiquitinate HAPSTR1, resulting in its own removal from chromatin. This would enable chromatin clearance to take place before the start of replication without the risk of interference with the replication machinery.

Given that cell cycle progression relies heavily on chromatin protein composition, it is not unexpected that HAPSTR1 loss triggers aberrant cell cycle dynamics, characterized by premature S phase entry, replication stress (consistent with recently reported results in U2OS cells) (*39*), and mitotic delay. In unperturbed cells, these effects seem to be tolerated through compensatory stress response pathways. However, under conditions of UPS inhibition, such cellular responses are inhibited, ultimately leading to cell death. Upon exacerbation of proteostatic stress by TAK-243 treatment, we observe in HAPSTR1 KO cells recruitment of heat shock proteins HSPB1 (HSP27) and HSPA1B (a member of the HSP70 family) to the chromatin. Heat shock proteins, traditionally associated with protein quality control, are increasingly acknowledged for their chromatin-associated functions and DNA repair—particularly HSPB1 and HSP70 proteins (*42*, *43*). HSPB1 has been shown to control cell-cycle progression and translation initiation to allow for DNA repair, while HSP70 contributes to DNA replication through facilitation of proliferating cell nuclear antigen (PCNA) loading on the chromatin (*44*). Moreover, HUWE1 also interacts with PCNA to alleviate replication stress (*45*). Thus, chaperone recruitment upon HAPSTR1 loss might reflect a broader cellular strategy to preserve genome integrity under ubiquitin signaling suppression. In this light, our transcriptomic and proteomic profiling of HAPSTR1-deficient cells reveals extensive remodeling of cell cycle and gene expression pathways that may aid in adaptation. For instance, we identify elevated levels of CDK6 and CDC6, both regulators of replication timing (*46*, *47*) and origin licensing (*48*), as part of the cellular response to HAPSTR1 deficiency. CDK6 is often mentioned interchangeably with CDK4 (“CDK4/6”) and perceived as redundant in many contexts, due to close sequence homology (71% overall and 94% in their ATP-binding pocket) and overlapping functions of these kinases in the cell cycle (*49*, *50*). Although some independent functions and regulation of CDK4 or CDK6 have been described (*46*), these differences remain underappreciated. We find that transcription of CDK4 and CCND remains unchanged in cells lacking HAPSTR1, providing evidence of CDK6-specific regulation. Collectively, cellular adaptations in response to HAPSTR1 ablation suggest that ubiquitin-dependent regulation of cell cycle proteins, including the DNA replication–origin regulator CDC6 (*51*, *52*) and the key S phase regulator CDK6 (*33*), helps to preserve cell proliferation despite the imbalances in chromatin proteostasis. These findings align with HUWE1-mediated degradation of CDC6 following DNA damage (*53*). In this light, changes brought about by HAPSTR1 loss may therefore signify an attempt to push cells through S phase and sustain DNA replication under conditions of impaired chromatin proteostasis. We uncovered a requirement of HAPSTR1 for integrity of the CDK6-RB-E2F1-CCNE-CDC6 replication axis and genome stability. This extends the literature where a C16orf72/Senataxin/BRCA1-dependent pathway was identified that suppress replication-associated R-loop accumulation (*41*).

Our findings diverge from the results of a TAK-243 genetic screen conducted in p53 wild-type AML cells (*16*). Using a less stringent false discovery rate (FDR) threshold of <0.2, only 2 of 33 synthetic-lethal genes identified in AML cells were among our 239 synthetic-lethal genes. These genes, the transcriptional repressor REST and DUB USP15, are potentially part of a broadly shared mechanism orchestrating cell proliferation via ubiquitin signaling pathways. The overlap between 33 genes conferring resistance in AML cells and 68 genes alleviating TAK-243 effects in RPE1-p53^−/−^ cells was limited to the transcriptional repressor BEND3. BEND3 loss has been linked to increased ABCG2 expression, which promotes drug efflux (*16*). Our observation of high synthetic lethality between ABCG2 and TAK-243 therefore suggests a shared mechanism through which BEND3 may mediate TAK-243 resistance in RPE1-p53^−/−^ cells. Nevertheless, beyond potential differences in adaptive changes or dropout events between the two screens, low numbers of overlapping hits likely reflect cell type–specific interplay between ubiquitin signaling and cell proliferation. This is exemplified by our findings that genetic ablation of the enigmatic protein HAPSTR1 is lethal under conditions of diminished ubiquitin signaling, whereas this was not observed in AML cells (*16*).

Together, we provide a valuable resource that contributes to our understanding of the interplay between the ubiquitin system and cell proliferation. Exploring one key candidate, HAPSTR1, we uncover a molecular pathway governing the coordination between chromatin proteostasis and cell cycle integrity. We show that HAPSTR1 loss evokes a dynamic adaptation of cell cycle control mechanisms through transcriptional and proteomic responses. Our findings support a buffering role for HAPSTR1 in chromatin proteostasis and cellular proliferation, echoing the role of its tissue-specific family member HAPSTR2 in stress signaling (*54*). However, under diminished UPS function, this buffering capacity is reduced, leading to breakdown in cell cycle integrity. This vulnerability may be particularly relevant in cancer, where levels of proteotoxic stress are intrinsically high due to heightened proliferative demands and genetic instability (*55*). Augmenting proteotoxic stress has therefore received extensive attention in the context of cancer therapy (*4*, *56*). In line with this, TAK-243 has shown substantial antitumor activity in preclinical cancer models (*14*, *57*–*59*) and is currently being evaluated in a clinical trial for leukemia treatment (NCT03816319). HAPSTR1 was previously described as an important fitness factor for the stress response of multiple cancer cell lines (*20*). Together with our findings on the synergy between ubiquitin signaling and HAPSTR1 loss, these considerations underscore important vulnerabilities of cancer cells and provide a foundation for further exploration toward therapeutic interventions in cancer.

## MATERIALS AND METHODS

### Plasmids

For the multicolor competition assays and generation of KO cell lines, HAPSTR1 and LacZ sgRNAs were cloned using oligo-duplex ligation into the lentiviral expression constructs: pLentiGuide-NLS-GFP-P2A-Puro or pLentiGuide-NLS-mCherry-P2A-Puro (*60*) (kind gift from S. Noordermeer). Sequences of the sgRNAs used in this study are included in table S2. The cDNA of HAPSTR1 was obtained from the MISSION TRC Human ORF Collection (Sigma-Aldrich, clone ID 03065) in the donor vector pDONR223. HAPSTR1-ΔA96 was generated via site-directed mutagenesis using the Q5 site-directed mutagenesis kit according to the manufacturer’s protocol (NEB, #E0554S). The oligonucleotides that were used are included in the key resource table, and the mutation was verified by DNA sequencing. Subsequently, HAPSTR1 was cloned into pDEST-C-term-Venus and both HAPSTR1 and HAPSTR1-ΔA96 were cloned into pDEST-N-term-eGFP using Gateway cloning. For fusions with an N-terminal V5 tag, HAPSTR1 cDNA was cloned into pcDNA3.1/nV5-DEST using standard Gateway technology (Invitrogen, #11791-100). Lentiviral doxycycline-inducible constructs of N-terminal GFP-fused HAPSTR1 were obtained as follows. GFP was cloned into pCW57.1 (Addgene, #41393) via restriction ligation and HAPSTR1 cDNA was inserted into this plasmid using Gateway cloning. The construct encoding His^10^-ubiquitin-IRES-puromycin resistance gene (His^10^-Ub) was published previously (*61*).

For small interfering RNA (siRNA)–mediated knockdown, SMARTpool ON-TARGETplus human C16orf72 (10 nM for 48 hours; Dharmacon, #L-031072-00-0020), HUWE1 siRNA (10 or 25 nM for 48 or 72 hours; Dharmacon, #L-007185-00-0050) and nontargeting sequences (Dharmacon, #D-001810-10-50) were transfected using RNAiMAX (Invitrogen, #13778150) in Opti-MEM (Gibco, #31985047).

### Reagents

Detailed information about reagents used in this study can be found in table S2.

### Cell culture and transductions

Cell lines—The human telomerase-expressing nontransformed epithelial retina cell line RPE1-hTERT-WT (female origin) was obtained from American Type Culture Collection (ATCC; #CRL-4000). RPE1-hTERT-p53^−/−^-PAC^−/−^-TetOnCas9 (referred to as RPE1-p53^−/−^ here) was previously described (*62*). RPE1 is a diploid genetic stable cell line well suited for and frequently used in genome-wide CRISPR-Cas9 KO studies. This cell line is deficient for p53 to enable identification of events beyond p53 induction. This cell line is also deficient for PAC, the puromycin *N*-acetyltransferase gene, because we use puromycin selection for sgRNAs. RPE1-hTERT-p53^−/−^-PAC^−/−^ was a kind gift from S. Noordermeer (*63*). All RPE1 cells were grown in Iscove’s modified Dulbecco’s medium (IMDM; Gibco) supplemented with 9% fetal bovine serum (FBS; Biowest) and 1% penicillin/streptomycin (Gibco). U2OS (ATCC, #HTB-96), HeLa (a kind gift from the laboratory of R. Medema at the Netherlands Cancer Institute), and human embryonic kidney (HEK) 293 cells (ATCC, #CRL-3216) were grown in Dulbecco’s modified Eagle’s medium (DMEM; Gibco). All cells were maintained in humidified incubators at 37°C and 5% CO_2_ and tested regularly for mycoplasma contamination and confirmed to be negative.

Lentiviral transduction—HEK293T cells were used to produce lentivirus in all cases. RPE1-hTERT-p53^−/−^-PAC^−/−^ cells stably expressing doxycycline-inducible GFP or GFP-HAPSTR1 were generated by lentiviral transduction. Lentiviral vectors were cotransfected with pCMV-VSVG, pMDLg-RRE, and pRSV-REV into HEK293T cells using polyethylenimine (PEI). After 24 hours, medium was changed to DMEM supplemented with 9% FBS/1% penicillin/streptomycin. 72 hours posttransfection, virus containing medium was collected, centrifuged at ×100*g*, and filtered with a 0.45-μm filter. Viral transductions were performed in DMEM supplemented with 9% FBS in the presence of polybrene (8 μg/ml), and transduced cells were selected and maintained with puromycin (1 μg/ml). GFP and GFP-HAPSTR1 expression were induced by doxycycline (100 and 500 ng/ml), respectively, 24 hours before experimentation. RPE1-p53^−/−^ cells stably expressing His^10^-Ub were generated by lentiviral transduction of a construct encoding for His^10^-Ub and a puromycin resistance gene separated by an internal ribosomal entry site (IRES). Cells were selected and maintained with puromycin (6 μg/ml). *HAPSTR1* gene KOs were generated in RPE1-p53^−/−^ cells and for HAPSTR1-KO.2, cells were transduced with lentivirus produced using third-generation virus packaging system and pLentiGuide-NLS-GFP-P2A-Puro backbone expressing HAPSTR1 sgRNA2. Transduced cells were selected using puromycin (2 μg/ml). After selection, the cells were treated with doxycycline (100 ng/ml; Sigma-Aldrich) to induce Cas9 expression for gene editing. Single clones were selected, and the gene editing of KO clone was confirmed by Sanger sequencing and immunoblotting. For HAPSTR1-KO.3, −8, −9, and −10, cells were transduced with HAPSTR1 sgRNA3, selected using puromycin (6 μg/ml), and Cas9 expression was induced by doxycycline (200 ng/ml), followed by single clone isolation using FACS sorting (BD FACSAria III Cell Sorter). Loss of protein expression was confirmed by immunoblotting for all KO clones.

### Drug treatment and synchronization

Ub E1 inhibition—RPE1-p53^−/−^ cells were treated with TAK-243 or dimethyl sulfoxide (DMSO) as indicated and imaged by time-lapse microscopy. Acquisition of phase images started at time of treatment on an Incucyte S3 Live-Cell Analysis System (Essen Bioscience) in a heated culture chamber (5% CO_2_ at 37°C) using IMDM with 9% FBS and antibiotics. The microscope was equipped with 10× phase contrast objective. Phase contrast images were acquired using the Incucyte software (Essen Bioscience) with 2-hour intervals for 96 hours. For quantitative analysis of confluency over time, the Incucyte software and the Prism software (GraphPad, v9.3.1) were used. Confluency was measured in nine images per well in technical triplicates and three independent experiments and plotted against the time from the start of treatment. Captured images were processed using Photoshop and Illustrator software (Adobe).

UPS inhibition—For inhibition of the ubiquitin E1 enzyme or the proteasome, cells were treated with TAK-243 (0.5 μM for 17 hours or as indicated; Active Biochem, #A-1384) or MG132, respectively (10 μM for 6 hours or as indicated; Sigma-Aldrich, #C2211-5MG).

Synchronization and release—RPE1-p53^−/−^, HAPSTR1-KO.2, and HAPSTR1-KO.10 cells were synchronized in mitosis by a 24-hour thymidine block (2.5 mM; Sigma-Aldrich, #T1895), followed by a nocodazole block of 17 hours (100 ng/ml; Sigma-Aldrich, #M1404-10MG). Mitotic cells were collected by mitotic shake-off, and a small fraction of cells were lysed [in 2% SDS, 1% NP-40, 50 mM tris (pH 7.5), and 150 mM NaCl] for *t* = 0. The remaining cells were released by washing and replated in fresh medium for *t* = 2 to 10 hours.

### CRISPR KO screen

Sample preparation*—*A total of 90 × 10^6^ cells, divided in three replicates, was transduced with the human pLCKO-TKOv3 CRISPR guide library (71,090 gRNAs; Addgene, #125517) (*64*) at a multiplicity of infection (MOI) of ∼0.25 in medium with polybrene (8 μg/ml) added. Twenty-four hours after transduction, transduced cells were selected on medium containing puromycin (6 μg/ml). After 72 hours of selection, each of the three replicate cell populations consisted of enough cells to take a *t* = 0 sample and seed untreated or untreated and TAK-243-treated populations at 400-fold library representation in the presence of doxycycline (200 ng/ml) to induce Cas9 expression. Cells were passaged every 3 days (untreated) or 3 to 4 days (TAK-243 treated) at 400-fold library representation for a total of ∼12 population doublings. A total of 30 × 10^6^ cells per condition per replicate was collected for genomic DNA (gDNA) extraction at *t* = 0 and after ∼12 population doublings.

gDNA was isolated using the QIAGEN Blood & Cell Culture DNA Maxi Kit (QIAGEN, #13362) and dissolved in a 2 mM tris-Cl (pH 9.0) and 0.5 mM EDTA buffer. Lentiviral inserts were amplified in 64 parallel 50-μl PCR reactions per sample, each reaction containing 3 μg of gDNA, 0.25 μl of 100 μM forward primer (AGGGCCTATTTCCCATGATTCCTT, IDT), 0.25 μl of 100 μM reverse primer (TCAAAAAAGCACCGACTCGG, IDT), and 25 μl of KAPA HiFi HotStart ReadyMix (Roche). Inserts were amplified by the following PCR program: 95°C (3 min), 28 cycles [98°C (20 s), 66°C (15 s), and 72°C (15 s)], 72°C (1 min), and 4°C (∞). Reactions of each sample were pooled, and Illumina adapters and index sequences were added in a second PCR reaction: 0.5 μl of the first PCR as a template, 16.5 μl of H_2_O, 4 μl of 10 μM forward primer {AATGATACGGCGACCACCGAGATCTACAC-[8–nucleotide (nt) Illumina i5 index sequence]-ACACTCTTTCCCTACCGACGCTCTTCCGATCTTGTGGAAGGACGAG GA*C*C*G, * = phosphorothioate bond, IDT}, 4 μl of 10 μM reverse primer [CAAGCAGAAGACGGCATACGAGAT-(8-nt reverse complement of Illumina i7 index sequence)-GTGACTGGAGTTCAGACGTGTGCTCTTCCGATC TATTTTAACTTGCTATTTCTAGCTCTAA*A*A*C, * = phosphorothioate bond, IDT], and 25 μl of KAPA HiFi HotStart ReadyMix. sgRNA sequences were amplified by the following PCR program: 95°C (3 min), 16 cycles [98°C (20 s), 66°C (15 s), and 72°C (15 s)], 72°C (1 min), and 4°C (∞). The PCR reactions were purified using the QIAquick PCR Purification kit (QIAGEN, #28104), equimolarly pooled, and sequenced using a standard SR50 run on an Illumina HiSeq 4000 (RPE1-p53^−/−^ screen) or a NovaSeq X Plus (HAPSTR1-KO screen). Sequencing reads were mapped to the TKOv3 library without allowing any mismatches, and the screen was analyzed using DrugZ (*65*).

Network analysis—Genes with a significant fold change of a combined FDR < 0.1 (four guides per gene) were identified as synthetic lethal. Significant hits were subjected to network analysis in Cytoscape (v 3.10.1) using STRING database, with a confidence cutoff score of 0.4. The following settings were applied: Each node presents one protein, and the edge width presents the interaction score. For generation of subcluster of interconnected proteins, the Cytoscape app MCODE was used with default parameters. Functional enrichment analysis was obtained in Cytoscape based on the STRING database with the humane proteome as background.

### Multicolor competition assay

A total of 40,000 RPE1-p53^−/−^ cells was infected at an MOI of ∼1.0 to ensure 100% transduction efficiency with lentivirus particles expressing either NLS-mCherry LacZ-sgRNA (a nontargeting control gRNA) or NLS-GFP gene of interest (GOI)–sgRNA targeting the GOI. Twenty-four hours after transduction, transduced cells were selected on medium containing puromycin (6 μg/ml). mCherry- and GFP-expressing cells were mixed 1:1 (7500 cells + 7500 cells) and plated in 12-well format 30 hours postselection. The following day, cells were treated with doxycycline (200 ng/ml) to induce expression of Cas9 and treated with TAK-243 (0.3 to 0.4 μM) or DMSO. During the course of the experiment, cells were subcultured when near confluency was reached. TAK-243–containing medium was replaced every 4 days. Cells were imaged for GFP and mCherry signal at the day of start of treatment (*t* = 0) and on days 4, 7, 11, 14, and 18 using the Incucyte S3 Live-Cell Analysis System (Essen Bioscience) with a 10× objective, taking 16 images per well. Counting of GFP^+^ and mCherry^+^ cells was performed using the Incucyte software. For quantitative analysis of the GFP^+^ fraction, the Excel (Microsoft) and Prism software (GraphPad, v9.3.1) were used. To assess gene editing efficiencies of the used sgRNAs, whole-cell lysates were isolated 6 days posttransduction and processed for immunolotting. Efficiency of indel formation was analyzed by PCR amplification, Sanger sequencing of the region surrounding the sgRNA targeting sequence, and TIDE analysis (*66*) on cDNA (HAPSTR1 sgRNA1) or gDNA (HAPSTR1 sgRNA2) isolated from samples 6 days posttransduction.

### AlphaFold structure prediction and complex modeling

Structural models of the HUWE1-HAPSTR1 complex were generated using AlphaFold3 (*67*). Because full-length HUWE1 is large and partially disordered, modeling was restricted to the deletion-mapped region (residues 2365 to 3090) together with full-length HAPSTR1. Multiple independent multimer predictions were run using default parameters without templates, and models were ranked by predicted local distance difference test and ipTM. Predicted complexes were visualized and analyzed in UCSF ChimeraX (*68*). Interfaces were inspected manually, and residues contributing to helix-helix packing were identified on the basis of proximity (<4 Å). Structural figures were prepared from the highest-confidence ranked model. To evaluate compatibility with the overall architecture of HUWE1, the predicted HUWE1 (3014 to 3049)–HAPSTR1 complex was superposed onto the experimentally determined full-length HUWE1 structure [PDB entry 7JQ9 (*23*)] using the tower motif as an alignment reference, and potential steric clashes were visually assessed.

### Flow cytometry

EdU incorporation—For analysis of DNA replication, RPE1-p53^−/−^ and HAPSTR1-KO.2 or HAPSTR1-KO.10 cells were pulsed with EdU (10 μM; Sigma-Aldrich, #900584) for 1 hour. After labeling, cells were detached by trypsinization, washed in 1% bovine serum albumin (BSA)–phosphate-buffered saline (PBS), and further processed for immunoblotting or fixed in 4% paraformaldehyde (PFA; 15 min) for flow-cytometry analysis. Fixed cells were washed in PBS containing 1% BSA and then permeabilized at room temperature (RT) for 15 min in PBS containing 1% BSA and 0.5% Triton X-100, and EdU was labeled with 1 μM AF647-Picolyl-Azide (Jena Bioscience, #CLK-1300-1) through Click-iT chemistry using 2 mM CuSO_4_ and 10 mM sodium ascorbate (Sigma-Aldrich, #A7631) for 30 min in the dark at RT. Cells were counterstained with 4′,6-diamidino-2-phenylindole (DAPI) (1 μg/ml of final concentration) in PBS containing 1% BSA, 0.1% NP-40, and ribonuclease A (RNase A; 100 μg/ml of final concentration) overnight in the dark at 4°C. Single color and unstained controls were taken along as negative controls to verify specificity of the staining. Flow-cytometry measurements were performed on the BD LSR II cell analyzer using FACSDiva (BD Biosciences). DAPI and EdU intensity were analyzed using the FlowJo software (v10.8.1).

Cell cycle analysis—For single cell GFP-HAPSTR1 detection, RPE1-hTERT-p53^−/−^-PAC^−/−^ cells stably expressing doxycycline-inducible GFP or GFP-HAPSTR1 were treated with 0.5 μM TAK-243 for 17 hours when indicated. For flow-cytometry measurement, cells were detached by trypsinization, washed in PBS containing 1% BSA, and further processed for immunoblotting or fixed in 4% PFA (15 min) for flow-cytometry analysis. Fixed cells were then permeabilized at RT for 15 min in PBS containing 1% BSA and 0.5% Triton X-100 and counterstained with DAPI (1 μg/ml of final concentration) in PBS containing 1% BSA and RNase A (100 μg/ml of final concentration) overnight in the dark at 4°C. Unstained controls were taken along as negative controls to verify specificity of the staining. Flow-cytometry measurement was done on the BD LSR II cell analyzer using FACSDiva (BD Biosciences). DAPI and GFP intensity along with cell cycle analysis (Watson Pragmatic algorithm) were analyzed using the FlowJo software (v10.8.1).

### DNA fiber assay

Following DMSO or TAK-243 treatment (17 hours, 0.5 μM), RPE1-p53^−/−^ and HAPSTR1-KO.2/KO.10 cells were pulse labeled with 25 μM 5-chloro-2′-deoxyuridine (CldU; Merck, #C6891) for 20 min and washed three times with PBS, followed by 20-min labeling with 250 μM 5-iodo-2′-deoxyuridine (IdU; Merck, #I7125). Labeled cells were harvested and resuspended in ice-cold PBS, of which 2 μl of cell suspension was spotted on a positively charged slide (VWR) and then horizontally incubated with 7 μl of lysis buffer [200 mM tris-HCl (pH 7.4), 50 mM EDTA, and 0.5% (w/v) SDS]. After 5 min, slides were tilted at ∼45°, allowing the drop to run by gravity. The DNA spreads were air dried, fixed in methanol/acetic acid (3:1; RT) for 10 min, and stored overnight at 4°C. Slides were rehydrated by rinsing three times with PBS, DNA was denatured with 2.5 M HCl for 1 hour at RT, and slides were washed four times with PBS. Antibody staining was performed at RT in the dark as followed. Slides were first blocked in 2% BSA in PBS/0.1% Tween 20 (PBST) for 1 hour and then stained for 2 hours with primary antibodies against CldU and IdU diluted in blocking buffer, washed three times with PBST, incubated with 2% formic acid (FA) for 10 min, and washed again three times with PBST. Slides were then incubated with secondary antibodies diluted in blocking buffer (anti-rat Alexa Fluor 555 and anti-mouse Alexa Fluor 488) for 1 hour and washed three times with PBST and once with blocking buffer. Slides were air dried for 1 hour, mounted with Aqua-Poly/Mount (Brunschwig), and conserved at 4°C in the dark until imaging. Images were acquired using a Zeiss Axio Imager.M2 widefield fluorescence microscope equipped with 40× Plan-Apochromat (1.4 numerical aperture) oil-immersion objectives (Zeiss) and an HXP 120 metal-halide lamp used for excitation. Images were recorded and analyzed with the ZEN 2012 (Blue edition, version 1.1.0.0) software and analyzed in ImageJ (*69*). Upon imaging, we selected isolated and good quality fibers, and through a tool in ImageJ, we distinguish the track length of the first label (in red) and the second label (in green). We then calculate the total length of the labels by adding them together and using a formula as in (*70*). We convert the micrometers obtained through our analysis in base pairs to obtain a measure of fork speed. Replication fork speed (in kilobases per minute) was calculated on the basis of the assumption that 1 μm of DNA fiber corresponds to 2.59 kb as previously shown (*70*). All measurements are normalized to wild-type (WT) condition (no treatment) to allow easy comparison between the different replicates.

For the analysis of origin firing, fibers, which have the following pattern, are identified: green-red-green-red-green. This pattern allows us to distinguish two different origins or replication that fired (*71*). In this case, we only perform one measurement, which is the IOD; in short, the distance between the middle parts of the two red sections, where we can hypothesize the origins are located. Similarly to before, we convert the measurement in micrometers to kilobases. To improve the interpretation of these data, we chose to extrapolate the number of origins from the IOD: For each measured IOD, we calculate how many origins would be required to replicate a region of 1 Mbp. This number gives us a representation of the number of origins that fire or are used.

### Immunofluorescence for γH2AX

Cells were grown on coverslip for 48 hours before the experiment and treated as indicated. Nuclei were preextracted with 0.5% Triton X-100 for 5 min on ice, carefully washed with PBS, and then fixed with 4% PFA for 10 min at RT in the dark. After fixation, coverslips were permeabilized by 0.5% Triton X-100 for 10 min at RT. Coverslips were blocked in 5% BSA in PBS overnight. Primary antibody [anti-γH2AX phospho-S139 clone JBW301 (05-636-I)] diluted 1:1000 in blocking buffer was incubated on coverslips in humid chamber in the dark for 1 hour, then coverslips were washed with PBS for 3 × 5 min and secondary antibody [goat anti-Mouse Alexa 555 (yellow) (A-21424) diluted 1:1000 in blocking buffer] was incubated on coverslips in humid chamber in the dark for 1 hour, and then coverslips were washed with PBS for 3 × 5min. Nuclei were counterstained with DAPI (0.1 μg ml^−1^) and then mounted onto glass slides with Polymount (Brunschwig, 18606).

### Time-lapse microscopy

For fluorescence microscopy, U2OS cells were plated in a glass-bottom 35-mm cell culture dish (ibidi), and when indicated, HUWE1 was knocked down using siRNA 48 hours before the start of acquisition. Cells were transfected with 0.4 μg of expression plasmids [enhanced GFP (eGFP)–HAPSTR1, HAPSTR1-Venus, or NLS-GFP] using Lipofectamine 2000 (Invitrogen, #11668019), followed by fluorescence time-lapse microscopy. When indicated, cells were treated with TAK-243 (0.5 μM) at the start of acquisition. For mitotic time-lapse microscopy, RPE1-p53^−/−^, HAPSTR1-KO.2, and HAPSTR1-KO.10 cells were plated in a four-compartment glass-bottom 35-mm cell culture dish (Greiner Bio-One). Acquisition of differential interference contrast (DIC) and fluorescence images started 48 hours after seeding cells or 24 hours after transfection on an Andor Dragonfly 500 microscope (Andor Technology) equipped with a heated humified culture chamber, an HCX PL APO 40×/1.30 oil DIC objective, and seven laser lines and appropriate emission filters to select specific fluorescence. Wide-field images were taken using the Fusion software (Andor Technology) with a Zyla sCMOS camera (Andor Technology) at 100-ms exposure times every 3 min for fluorescence microscopy and every 2 min for mitotic time-lapse microscopy. For quantitative analysis of degradation, ImageJ software, Excel (Microsoft), and Prism software (GraphPad, v9.3.1) were used. Signal intensities were measured in a defined area containing the cell of interest and normalized against the intensity at time point *t* = 0, nuclear envelope breakdown (NEB), as indicated. The intensity of fluorescence was plotted against the time from the start of NEB. Background intensity was subtracted from each measurement. Captured images were processed using Photoshop and Illustrator software (Adobe).

### Chromatin fractionation

Sample preparation—Chromatin-bound proteins were purified as followed with all steps performed on ice. Following DMSO or TAK-243 treatment (17 hours, 0.5 μM), cells were harvested by trypsinization and washed in ice-cold PBS. For total protein lysates, a small fraction of cells was lysed in SNTBS lysis buffer [2% SDS, 1% NP-40, 50 mM tris-HCl (pH 7.5), and 150 mM NaCl] and heated to 99°C for 10 min. The remaining cells were resuspended in buffer A [10 mM Hepes, 10 mM KCl, 1.5 mM MgCl_2_, 10% glycerol, 340 mM sucrose, 1 mM dithiothreitol (DTT; Sigma-Aldrich, #D0632), 1 mM *N*-ethylmaleimide (Sigma-Aldrich, #E3876), protease inhibitors (cOmplete Mini EDTA-free protease inhibitor cocktail, Sigma-Aldrich, #11836170001), and phosphatase inhibitors (4 mM sodium fluoride and 10 mM β-glycerophosphate)] and incubated with 0.1% Triton X-100 for 8 min. The supernatant was separated from the pellet by centrifugation at 1300*g* for 5 min at 4°C and subsequently centrifuged at 20,000*g* for 15 min at 4°C to collect the cytoplasmic fraction. The pellet was washed twice with buffer A, lysed in buffer B (3 mM EDTA, 0.2 mM EGTA, 1 mM DTT, and protease and phosphatase inhibitors) for 30 min, and centrifuged at 1700*g* for 5 min at 4°C. Next, the pellet was washed twice with buffer B, and this chromatin fraction was lysed in SNTBS and heated to 99°C for 15 min. A total of 9 μg of chromatin fraction was loaded onto NuPAGE 4 to 12% Bis-Tris Gels (Novex, Invitrogen, #NW04127BOX) and run for 4 min at 150 V, and the excised gel slices were subjected to automated in-gel digestion through the AssayMAP Bravo Platform (Agilent Technologies) following an in-house digestion protocol. Gel bands were first washed with 200 μl of both acetonitrile (ACN) and 100 mM ammonium bicarbonate (ABC), pH 8.4, followed by reduction using 200 μl of 10 mM DTT in 10 mM ABC, pH 8.4, at 60°C for 30 min, and alkylation using 200 μl of 55 mM iodoacetamide in 10 mM ABC, pH 8.4, at RT for 30 min. Digestion took place overnight at 37°C with 50 μl of a trypsin (porcine, sequencing grade, modified; Merck) solution containing 12.8 ng/μl in 25 mM ABC, pH 8.4. Peptides were extracted twice using a total volume of 150 μl of ACN/water [80/20 (v/v)] containing 1.0% FA.

MS acquisition—Samples were dissolved in water/FA [100/0.1 (v/v)] and subsequently analyzed by online C18 nano–high-performance liquid chromatography (HPLC) tandem MS (MS/MS) with a system consisting of a Vanquish Neo and an Orbitrap Astral mass spectrometer (Thermo Fisher Scientific). The Vanquish Neo was operated in trap and elute mode. Samples were injected onto a cartridge precolumn (300 μm × 5 mm, C18 PepMap, 5 μm, 100 Å) and eluted via a homemade analytical nano-HPLC column [21 cm × 75 μm; Reprosil-Pur C18-AQ 1.9 μm, 120 Å (Dr. Maisch, Ammerbuch, Germany)]. The gradient was run from 2 to 40% solvent B [20/80/0.1 of water/ACN/FA (v/v)] in 20 min. The nano-HPLC column was drawn to a tip of ∼10 μm and acted as the electrospray needle of the MS source. The mass spectrometer was operated in DIA mode at a resolution of 240,000 in MS1, with a scan range of 380 to 980 Th at an automatic gain control (AGC) value of 500% and a maximum fill time of 3 ms. MS was recorded at a higher-collisional dissociation (HCD) collision energy of 25%, with a 2-Th isolation window, and a maximum fill time of 5 ms with recording of the MS2 spectrum in the Astral. A lock mass correction on the background ion mass/charge ratio (*m/z*) = 202.0777 was used.

Data analysis—DIA raw data were processed using Proteome Discoverer version 3.2 (Thermo Fisher Scientific), using the CHIMERYS mode with INFERYS 4.7.0 as the prediction model, and with default settings. In short, the settings were the following: trypsin as the enzyme (max two missed cleavages), a peptide length between 7 and 30, a charge between 1 and 4, and a fragment tolerance of 20 ppm. Oxidation on methionine was set as a dynamic modification and carbamidometyl on cysteine as a fixed modification. FDR was set to 1%. Quantitation was done in MS Apex mode. The canonical human database was used. The MS proteomics data have been deposited to the ProteomeXchange Consortium via the PRIDE (*72*) partner repository with the dataset identifier PXD064507.

### His^10^-Ub purification

For detection of ubiquitinated HAPSTR1, RPE1-p53^−/−^ His^10^-Ub cells were transiently transfected with a V5-HAPSTR1 construct using PEI. For detection of ubiquitinated CDK6, RPE1-p53^−/−^ cells stably expressing His^10^-Ub were used along with HAPSTR1-KO clones 2 and 10 that were transduced with lentivirus expressing His^10^-Ub 72 hours before processing. His^10^-Ub–conjugated proteins were purified as previously described (*73*). Briefly, cells were collected by scraping in ice-cold PBS. For total protein lysates, a small fraction of cells was lysed in SNTBS lysis buffer [2% SDS, 1% NP-40, 50 mM tris-HCl (pH 7.5), and 150 mM NaCl]. The remaining sample was lysed in 6 M guanidine-HCl, 100 mM Na_2_HPO_4_/NaH_2_PO_4_ (pH 7.8), and 10 mM tris-HCl (buffered at pH 7.8). Cell lysates were sonicated (Misonix Sonicator 3000) and supplemented with 50 mM imidazole (pH 8) and 5 mM β-mercaptoethanol. Samples were incubated with nickel-nitrilotriacetic acid (Ni-NTA) agarose beads (QIAGEN) overnight at 4°C and washed and eluted in LDS sample buffer (Thermo Fisher Scientific, #NP0007), supplemented with 100 mM DTT and 500 mM imidazole, pH 7, at 99°C for 10 min.

### Endogenous ubiquitin purification using UbiSite methodology

Sample preparation—Enrichment and MS of endogenous ubiquitin substrates in RPE1-p53^−/−^ (five replicates) and HAPSTR1-KO.2 (five replicates) cells using UbiSite technology were performed as previously described (*30*). Briefly, cells were treated with MG132, washed twice with ice-cold PBS, and scraped and lysed in lysis buffer (8 M guanidine hydrochloride in 50 mM Hepes solution, pH 8.5). After sonication for 30 s, lysates were centrifuged at 16000 RCF for 15 min, and protein concentration of each condition’s replica was measured with BCA Kit (Pierce, Thermo Fisher Scientific, #PI23227). Proteins were subjected to reduction with DTT (2 mM for 30 min at RT), alkylation with chloroacetamide (11 mM for 30 min at RT in the dark), and digestion with LysC enzyme (Wako) overnight (enzyme:protein ratio was 1:100). Peptides mixture was purified using C18 cartridge (Waters, #WAT051910) and lyophilized for 24 hours. The LysC-digested peptides were dissolved in ice-cold IAP buffer [50 mm Mops-NaOH (pH 7.6), 10 mm Na_2_HPO_4_, and 50 mm NaCl] in the presence of 0.1% Triton X-100. After brief centrifugation (16000 RCF for 5 min), peptide concentration was measured with a NanoDrop (Implen, NanoPhotometer). Three milligrams of peptides from each replica was mixed with 30 μl of UbiSite antibody-agarose beads (50% slurry) [the UbiSite antibody was bound and cross-linked to agarose beads as described earlier (*30*)] and incubated for 4 hours at 4°C slowly rotating (10 rpm). After incubation, the beads were washed with 1 ml of ice-cold IAP buffer, and captured peptides were eluted three times with 0.1% trifluoroacetic acid. The eluted peptides were further neutralized with Hepes buffer (100 mM, pH 8.0) and digested with 100 ng of trypsin at 37°C for 12 hours.

MS acquisition—The complex mixtures of peptides were purified using StageTips (*74*), spiked with iRT standard peptides (Biognosys AG, Switzerland), and subjected to nano-LC MS/MS analysis using an EASY-nLC 1000 ultrahigh-pressure system (Thermo Fisher Scientific) connected online with the Orbitrap Exploris 480 mass spectrometer (Thermo Fisher Scientific). Peptides mix from each replica were loaded into a fused-silica column home packed with 1.9-μm Reprosil C18 beads (Dr-Maisch, #r119.b9) in solvent A (0.5% acetic acid). Peptides were eluted with a gradient of solvent B (80% acetonitrile and 0.5% acetic acid) from 5 to 95% at flow rate of 250 nl/min. Data were acquired for 120 min using DIA method with the following settings: mass range of 350 to 1400 *m/z*, profile data type, normalized AGC target of 300% with 120,000 resolution, and 45 ms of maximum injection time for MS1 scans. DIA setup for 56 windows with 12 Da and overlap of 1 Da was used. Precursor mass range was 361 to 1033 *m/z*. Acquired scans had normalized AGC target value set at 1000%, Orbitrap resolution was 30,000, and maximum injection time was 54 ms. The collision energy was set at 28%.

Data analysis—Raw MS files were analyzed using Spectronaut (version 18.1.230626.50606) using the Direct DIA approach and the “Pulsar” search engine with the following parameters: Enzymes for protein digestion were trypsin with two missed cleavages and an exclusion for cleavage before proline; the minimum peptide length was seven residues. PSM, peptide, and protein group FDRs were set to 0.01, and PTM localization filter was active with minimum localization threshold set to 0.75. A UniProt database (reviewed Swiss-Prot from October 2019 of *Homo Sapiens*) with 20,351 entries was used for the Pulsar’s search. Acetyl (protein N terminus), oxidation (M), and ubiquitination (diGly on K) were set as variable peptide modifications, whereas carbamidomethyl (C) was set as fixed peptide modification. Cross-run normalization was set as “True” with default row selection and automatic function for normalization strategy. The MS proteomics data have been deposited to the ProteomeXchange Consortium via the PRIDE (*72*) partner repository with the dataset identifier PXD050046.

The Spectronaut PTM output table was filtered in Perseus (v2.0.7.0). Data were log_2_ transformed and filtered for identification in at least four of five replicates of RPE1-p53^−/−^ or HAPSTR1-KO.2. Missing values were imputed from the lower end of the normal distribution (default settings). A two-sided student’s *t* test with permutation-based FDR cells at 0.05 FDR (*q* value) and *S*_0_ = 0.1 was used for statistical analysis (table S4).

### MS–based interactomics

Sample preparation—For immunoblot analysis, HeLa cells were transiently transfected with eGFP-HAPSTR1 or eGFP-HAPSTR1-ΔA96 constructs using PEI and harvested in PBS using a cell scraper 1 day posttransfection. For MS analysis, RPE1-p53^−/−^-PAC^−/−^ cells stably expressing dox-inducible eGFP, eGFP-HAPSTR1, or eGFP-HAPSTR1-ΔA96 were treated with TAK-243 or DMSO when indicated and harvested by scraping in PBS. A small fraction of HeLa or RPE1-p53^−/−^-PAC^−/−^ total lysates were lysed in SNTBS and processed for immunoblotting, and the remaining cell pellets were lysed in IP-lysis buffer: 20 mM tris (pH 7.5), 150 mM NaCl, 10% glycerol, and 0.5% Triton X-100, supplemented with bezonase nuclease (Millipore, #70746), protease inhibitor cocktail (Sigma-Aldrich, #11697498001), and phosphatase inhibitors (β-glycerophosphate, NaVO_3_, NaF, Sigma-Aldrich). Lysates were treated with benzonase nuclease for 30 min on ice and cleared by centrifugation (14,000 rpm for 15 min at 4°C). Protein levels were measured and equalized using standard Bradford assay (Bio-Rad, #500-0205). Cleared lysate was incubated with GFP-Trap agarose beads (ChromoTek, #gta-20) for 2 hours while tumbling at 4°C. Beads were washed four times with IP-lysis buffer (last two washes without protease and phosphatase inhibitors). Elution of the immunoprecipitated proteins from HeLa cells was carried out by boiling for 10 min at 99°C in 1× LDS sample buffer (Thermo Fisher Scientific, #NP0007) and processed for immunoblotting. Beads incubated with RPE1-p53^−/−^-PAC^−/−^ cells were washed five times with 50 mM (NH_4_)_2_CO_3,_ digested overnight with 0.5 μg of trypsin in 50 mM (NH_4_)_2_CO_3_ at 37°C under shaking, and processed for MS analysis. Digested peptides were separated from the beads over 0.45-μm filter columns (Millipore, #UFC30HV00) prewashed with 50 mM (NH_4_)_2_CO_3._ Peptides were desalted and concentrated using in-house–assembled StageTips with three stacked C18 disks (3M, #WAT051910), as previously described (*74*). Peptides were eluted in 32.5% ACN (Sigma-Aldrich, #34998) in 0.1% FA (Sigma-Aldrich, #09676), vacuum dried, and dissolved in 0.1% FA.

LC-MS/MS—GFP-Trap samples were run on a Q Exactive Orbitrap mass spectrometer (Thermo Fisher Scientific) connected to an EASY-nLC 1000 system (Proxeon). Digested peptides were separated by a fritted fused-silica capillary (25-cm *L* × 75-μm inside diameter × 360-μm outer diameter; MS Wil, #ICT36007515F-25-5) in-house packed with 1.9-μm C18 beads (Dr. Maisch). The gradient length was 60 min from 2 to 38% solvent B (80% ACN/0.1% FA). Every gradient was followed by a 5-min increase to 95% solvent B and back to 0% ACN in 0.1% FA for chromatography column reconditioning. The mass spectrometer operated in positive polarity mode, using a 2.3-kV potential with the capillary heated to 250°C. Data-dependent acquisition mode with a top-10 method facilitated automatic switching between full-scan MS and MS/MS scans. Full scan MS spectra were acquired at a resolution of 70,000, an AGC target value of 3 × 10^6^, and a scan range of 400 to 1400 *m/z*. Precursors were fragmented by HCD with a normalized collision energy of 25%. MS/MS were recorded with a resolution of 17,500 and an AGC target value of 1 × 10^5^. Precursor ions selected for MS/MS analysis were subsequently dynamically excluded from MS/MS analysis for 60 s. Precursors with a charge of 1 or >6 were excluded from triggering MS/MS events. Maximum injection times for MS and MS/MS were set at 20 and 60 ms, respectively.

Data analysis—Raw MS files were analyzed using MaxQuant (v.2.1.3.0) with a search against an in silico digested reference proteome for *Homo Sapiens* (UniProt; reviewed, canonical, downloaded 27 August, 2022). Database searches were performed with default settings apart from the following modifications: Label-free quantification (LFQ), the match-between-runs feature, and match unidentified features were enabled (default settings). The MaxQuant output table “ProteinGroups” was further processed using Perseus (v2.0.7.0): Proteins identified as contaminants were filtered out, LFQ intensities were log_2_ transformed, only proteins identified in all replicates of at least one group were included, and missing values were imputed. Significant interactors were identified by a two-sided *t* test (*P* < 0.05, *S*_0_ = 0.1), and volcano plots were generated using VolcaNoseR (*75*). The MS proteomics data have been deposited to the ProteomeXchange Consortium via the PRIDE (*72*) partner repository with the dataset identifiers PXD049697 and PXD074872.

### Immunoblotting

Cells were harvested in ice-cold PBS using a cell scraper or trypsinization and washed once with PBS by centrifugation. Cell pellets were lysed for 10 min at 99°C in lysis buffer [2% SDS, 1% NP-40, 50 mM tris (pH 7.5), and 150 mM NaCl], unless stated otherwise. Lysates were cleared by centrifugation (14,000 rpm for 15 min at RT). Protein levels were measured and equalized using the Pierce BCA Protein Assay (Thermo Fisher Scientific, #23227) on a NanoDrop 1000 spectrophotometer, and samples prepared in LDS sample buffer were supplemented with DTT. Protein was separated on NuPAGE 4 to 12% Bis-Tris Gels and blotted on Amersham Protran Premium 0.45-μm nitrocellulose blotting membranes (GE Healthcare, #732-0591) using Mini Gel Tanks (Invitrogen). Blocking of membranes and antibody incubation was done in PBS containing 0.05% Tween 20 and 8% milk (ELK, Campina). Primary antibody incubation was performed overnight at 4°C, and secondary antibody incubation was performed for 2 to 5 hours at RT. For detection of phosphorylated proteins, blocking was done in tris-buffered saline (TBS) containing 0.05% Tween 20 and 8% milk, and antibody incubation was done in TBS containing 0.05% Tween 20 and 5% BSA. For chemiluminescent detection of horseradish peroxidase activity from antibodies, we use (max) Clarity Western ECL Substrate (Bio-Rad, #170-5060). Analysis was done using ChemiDoc Imaging System (Bio-Rad) or iBright (Thermo Fisher Scientific). Captured images were processed using Photoshop and Illustrator software (Adobe). ImageJ (v1.53t) or iBright analysis software (Thermo Fisher Scientific) was used for signal quantification of protein bands.

### Quantitative PCR and RNA sequencing

Sample preparation—RNA for qPCR or total RNA sequencing (RNA-seq) was isolated using the SV Total RNA Isolation System (Promega, #Z3100) according to the manufacturer’s protocol. qPCR was performed using FastStart Universal SYBR Green Master (Roche Diagnostics, #4913914001) in a C1000 Touch Thermal Cycler (Bio-Rad Laboratories). The relative expression of target genes was determined and corrected in relation to the housekeeping genes *ARP*, *CAPNS1*, *RPS11*, *SRPR*, and *TATA box–binding protein* (oligonucleotide sequences are shown in the key resource table).

Data analysis RNA-seq—Total RNA was isolated as described above, and TruSeq Stranded Total RNA Ribo-Zero Gold [ribosomal RNA (rRNA) depleted] was performed at Macrogen. Libraries were sequenced with 150-bp paired-end reads using the NovaSeq X Platform. Preprocessing, quality control, and deduplication were done using fastp v0.20.1 (*76*, *77*) with parameter --qualified_quality_phred 30. The reads were mapped to the hg38 reference genome (GENCODE release 47) using STAR aligner v2.7.0f (*78*) using default parameters with the following exceptions: “--sjdbOverhang 149,” “--winAnchorMultimapNmax 200,” and “--outFilterMultimapNmax 100.” Quantification was done by featureCounts v2.0.1 (*79*), using the GENCODE v47 annotation. Statistical analysis was done using DESeq2 v1.42.1 (*80*) (R package). Genes were considered differentially expressed by *P* < 0.05.
