# Supplementary material for: Chemogenetic ubiquitin E1 inhibitor TAK-243 CRISPR screen uncovers HAPSTR1/HUWE1 axis in chromatin proteostasis and cell cycle control
Source: Sci Adv. 2026 Oct 16;12(42):eaea3388. doi: 10.1126/sciadv.aea3388 (PMC13674506; doi:10.1126/sciadv.aea3388)
Supplement: Supplementary file 1 [file sciadv.aea3388_sm.pdf]

Science Advances
AAAS

# Supplementary Materials for

## Chemogenetic ubiquitin E1 inhibitor TAK-243 CRISPR screen uncovers HAPSTR1/HUWE1 axis in chromatin proteostasis and cell cycle control

Nila K. van Overbeek *et al.*

Corresponding author: Ilana Berlin, i.berlin@lumc.nl; Alfred C. O. Vertegaal, vertegaal@lumc.nl

*Sci. Adv.* **12**, eaea3388 (2026)
DOI: 10.1126/sciadv.aea3388

**The PDF file includes:**

Figs. S1 to S11
Tables S1 and S2
Legends for data S1 to S5

**Other Supplementary Material for this manuscript includes the following:**

Data S1 to S5

A

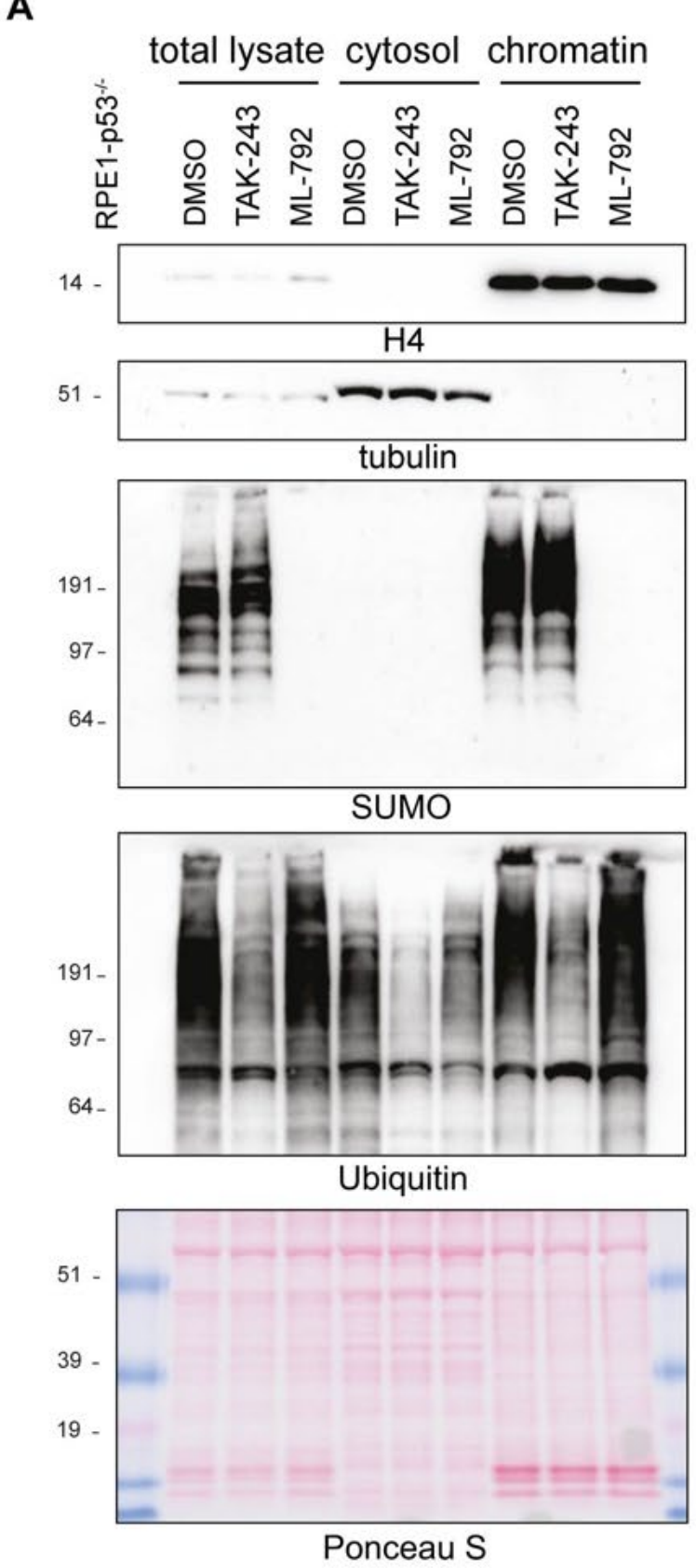


C

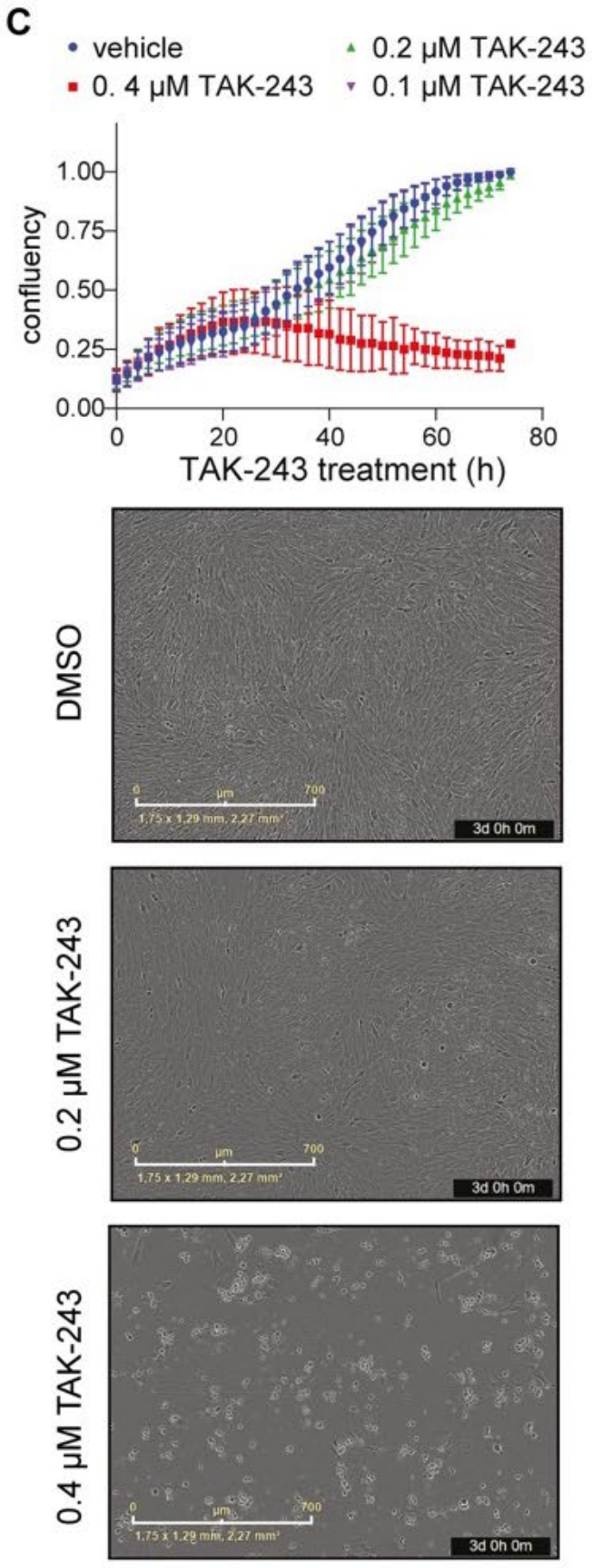


D

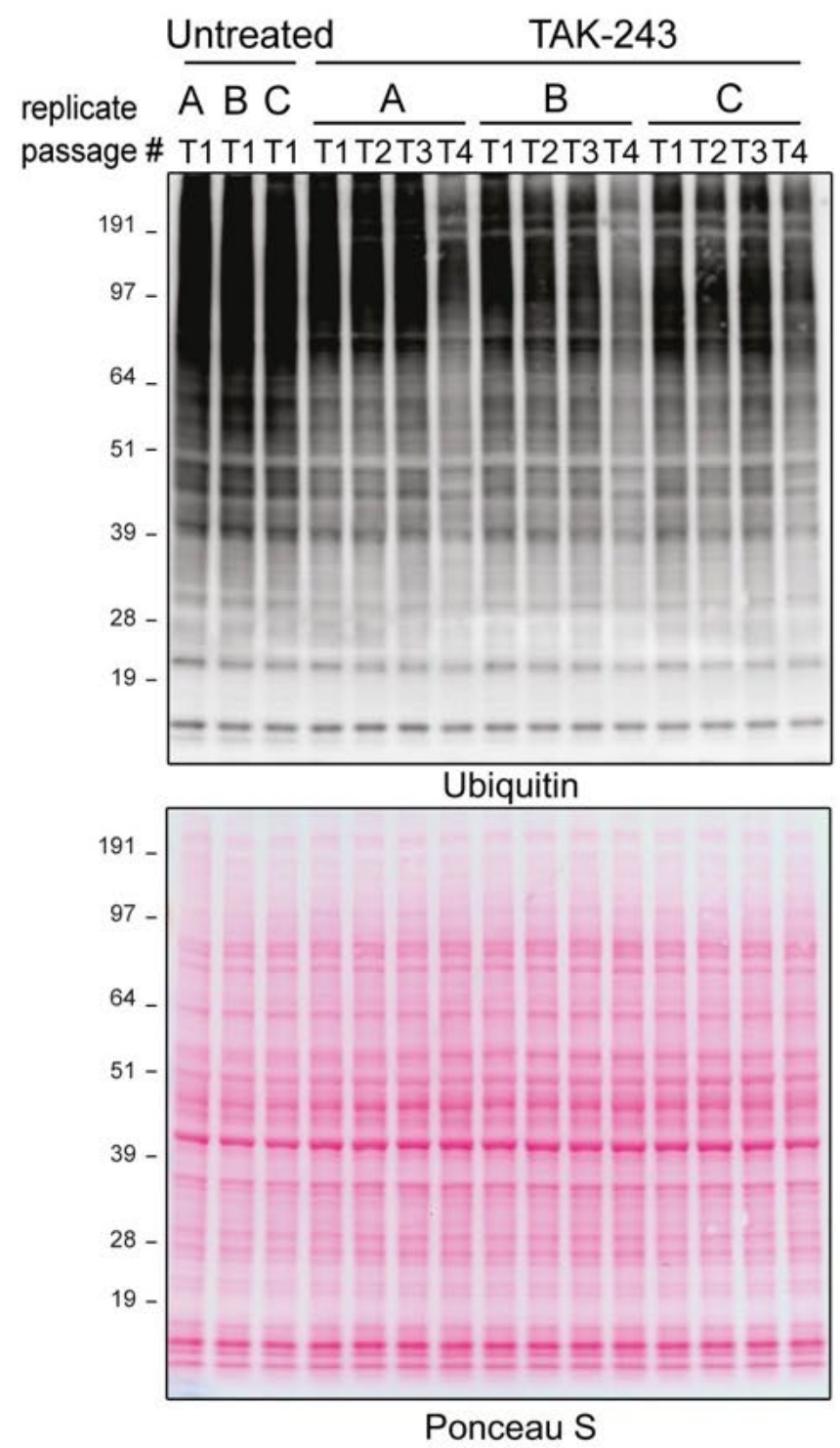


B

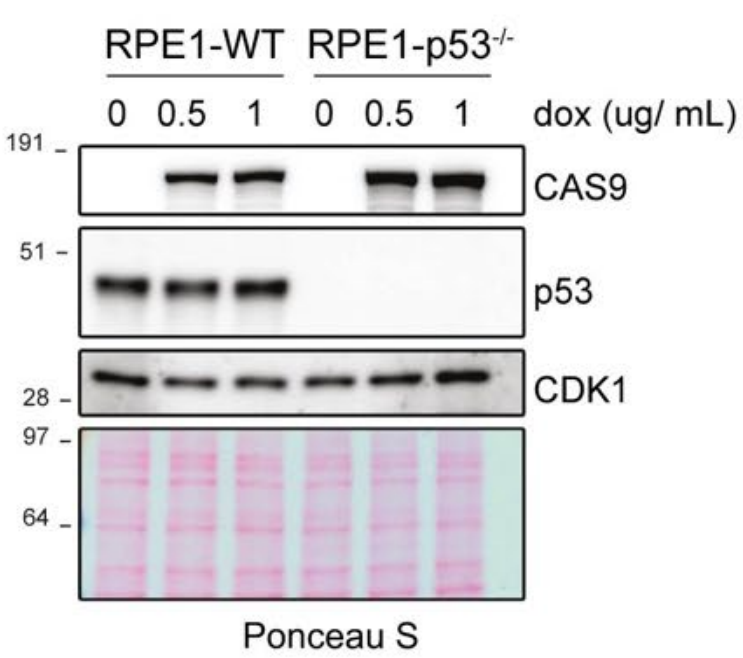


E

| | Sensitizing hits | FDR | Resistance hits | FDR |
|---|---|---|---|---|
| 1 | ABCG2 | 5.17E-57 | TSC2 | 7.97E-26 |
| 2 | CSNK1A1 | 4.72E-43 | PDCD10 | 9.82E-25 |
| 3 | C16orf72 | 2.32E-35 | PTEN | 6.18E-24 |
| 4 | TMEM208 | 2.25E-31 | TSC1 | 1.19E-20 |
| 5 | HIRA | 1.22E-27 | ACVR1B | 3.38E-17 |
| 6 | MIB1 | 1.37E-23 | TAOK1 | 5.82E-16 |
| 7 | FKBP8 | 2.56E-23 | FRYL | 9.23E-13 |
| 8 | CUL3 | 1.49E-22 | ACVR2A | 6.90E-11 |
| 9 | PCBP1 | 7.32E-21 | CAB39 | 7.55E-11 |
| 10 | ARMC5 | 8.14E-21 | KIRREL | 4.59E-10 |
| 11 | ACTR6 | 3.93E-19 | ZNF827 | 4.59E-10 |
| 12 | SOCS3 | 3.31E-18 | CCM2 | 5.33E-09 |
| 13 | SMARCB1 | 1.28E-16 | CEBPB | 8.15E-09 |
| 14 | PPP4C | 5.97E-15 | GNAS | 1.91E-08 |
| 15 | AIP | 5.85E-14 | ZFX | 1.96E-08 |
| 16 | CCNC | 8.90E-14 | RNF111 | 1.39E-07 |
| 17 | UBE2L3 | 2.07E-12 | BEND3 | 2.15E-07 |
| 18 | HSF1 | 3.93E-12 | SMAD4 | 8.47E-07 |
| 19 | PPP1R8 | 7.74E-12 | KEAP1 | 4.97E-06 |
| 20 | AP2S1 | 7.59E-11 | KCTD5 | 1.32E-05 |

**Figure S1. Drug sensitivity analysis to TAK-243 and top significant hits of the TAK-243 CRISPR KO screen (related to Figure 1).**

**A** TAK-243 does not inhibit SUMOylation. RPE1-p53$^{-/-}$ cells were treated with DMSO, TAK-243 (0.5 µM, 17h) or ML-792 (1 µM, 17h). Total lysates as well as cytosolic and chromatin fractions were analyzed by immunoblotting using antibodies directed against endogenous H4, tubulin, SUMO-2/3 and ubiquitin. Ponceau S staining was used as loading control. Data shown are representative of n=4 independent experiments.

**B** Cas9 induction. Representative immunoblots of lysates from asynchronous RPE1-wildtype (WT) and RPE1-p53$^{-/-}$ cells treated with doxycycline for two days, as indicated (CDK1 used as loading control).

**C** TAK-243 drug sensitivity analysis. Plot of average confluency (mean ± SD, n= 3 independent experiments) over time (h) of RPE1-p53$^{-/-}$ cells treated with DMSO (vehicle), 0.2 µM or 0.4 µM TAK-243, measured every 2h for 72h by automated time-lapse microscopy. Representative images of cells collected at t=72h are shown alongside, scale bar = 700 µm.

**D** Representative immunoblots against ubiquitin of lysates from RPE1-p53$^{-/-}$ cells taken at the moments of passaging during the chemogenetic CRISPR KO screen.

**E** Table of top 20 significant hits (resistance and sensitizing) in TAK-243 CRISPR KO screen.

A

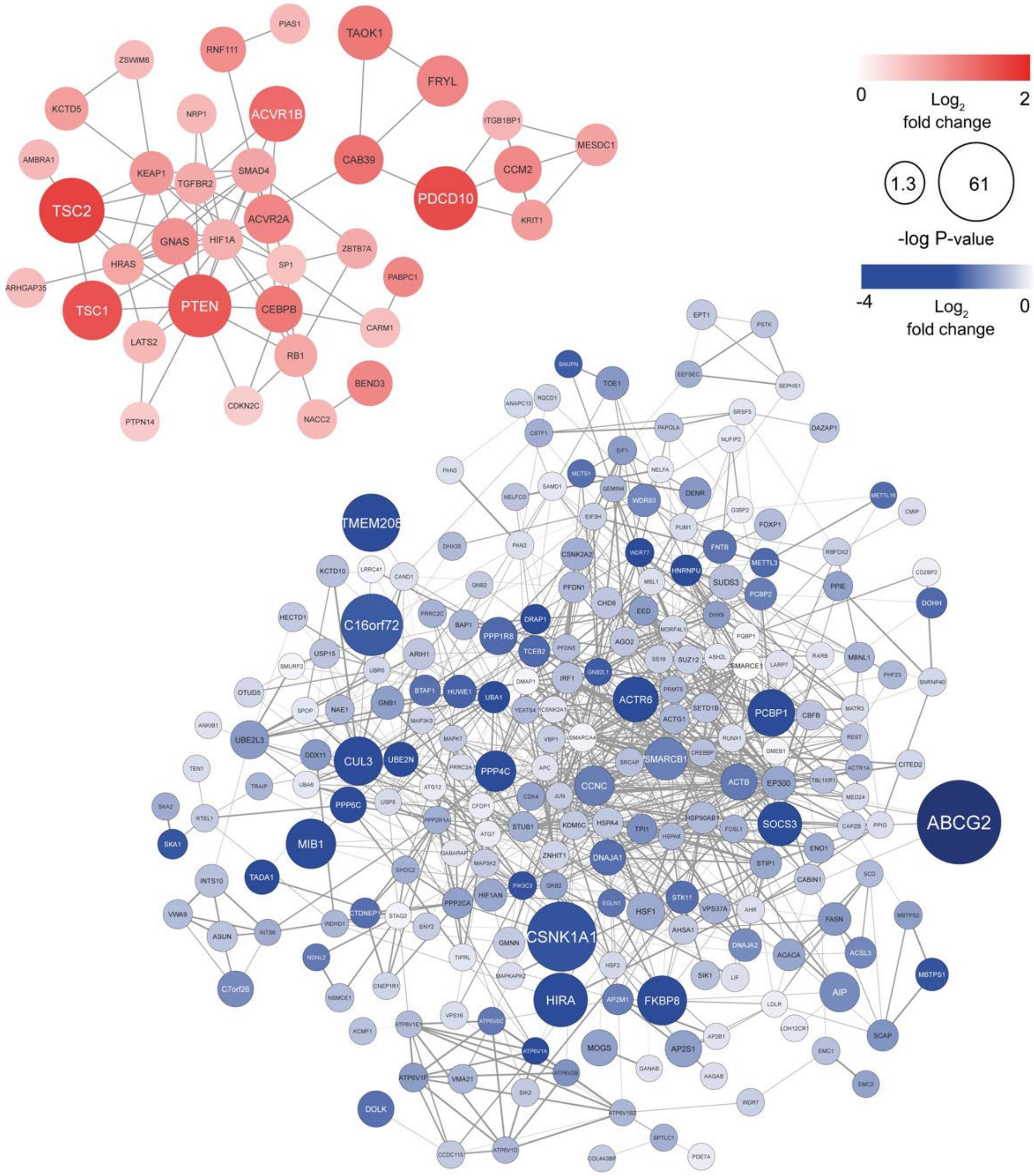


B

MCODE cluster generation

| | category | term name | description | FDR value | # genes | genes |
|---|---|---|---|---|---|---|
| 1 | Reactome Pathways | HSA-3247509 | Chromatin modifying enzymes | 1.26E-11 | 12 | SMARCB1, EP300, EED, REST, PRMT5, SMARCE1, MORF4L1, ASH2L, ACTB, SMARCA4, TBL1XR1, MSL1 |
| 2 | GO Molecular Function | GO:0046961 | Proton-transporting ATPase activity, rotational mechanism | 1.58E-15 | 7 | ATP6V1D, ATP6V1E1, ATP6V1A, ATP6V1B2, ATP6V0C, ATP6V1F, ATP6V0B |
| 3 | GO Biological Process | GO:0043486 | Histone exchange | 3.90E-03 | 3 | ACTR6, ZNHIT1, DMAP1 |
| 4 | GO Biological Process | GO:0090181 | Regulation of cholesterol metabolic process | 3.78E-09 | 5 | SCAP, FASN, SCD, LDLR, ACACA |
| 5 | Reactome Pathways | HSA-6807505 | RNA polymerase II transcribes snRNA genes | 2.53E-06 | 4 | ASUN, INTS6, INTS10, VWA9 |
| 6 | KEGG Pathways | hsa04912 | GnRH signaling pathway | 7.30E-04 | 4 | MAPK7, MAP3K3, JUN, MAP3K2 |
| 7 | Reactome Pathways | HSA-3247509 | Chromatin modifying enzymes | 6.30E-04 | 6 | WDR77, CDK4, CREBBP, SUZ12, KDM5C, SETD1B |
| 8 | GO Biological Process | GO:0007077 | Mitotic nuclear envelope disassembly | 1.83E-02 | 2 | CNEP1R1, CTDNEP1 |
| 9 | GO Molecular Function | GO:0003743 | Translation initiation factor activity | 2.10E-03 | 3 | DENR, MCTS1, EIF1 |
| 10 | GO Biological Process | GO:0006261 | DNA-dependent DNA replication | 3.00E-03 | 3 | RTEL1, WDHD1, DDX11 |
| 11 | GO Biological Process | GO:0000086 | G2/M transition of mitotic cell cycle | 9.80E-04 | 3 | HAUS3, HAUS8, HAUS1 |
| 1 | GO Biological Process | GO:0001932 | Regulation of protein phosphorylation | 5.10E-04 | 9 | ACVR2A, CDKN2C, SMAD4, ITGB1BP1, PTEN, LATS2, PDCD10, HRAS, ACVR1B |
| 2 | GO Molecular Function | GO:0061629 | RNA polymerase II-specific DNA-binding transcription factor binding | 7.20E-04 | 4 | RB1, CEBPB, SP1, HIF1A |

**Figure S2. Significant hits from the TAK-243 CRISPR KO screen (related to Figure 1).**
**A** STRING interaction network (confidence cut-off score of 0.4) of significant hits (FDR<0.1), causing resistance (red) or increasing sensitivity to TAK-243 (blue). The size of the node represents the -log p-value and the $log_2$ fold change vs DMSO is color coded.
**B** Clusters of interconnected proteins were generated using MCODE (default settings). The table shows the functional enrichment terms of the 11 clusters within the protein network of hits increasing sensitivity (blue) and of the 2 clusters within the protein network of hits causing resistance to TAK-243 (red).

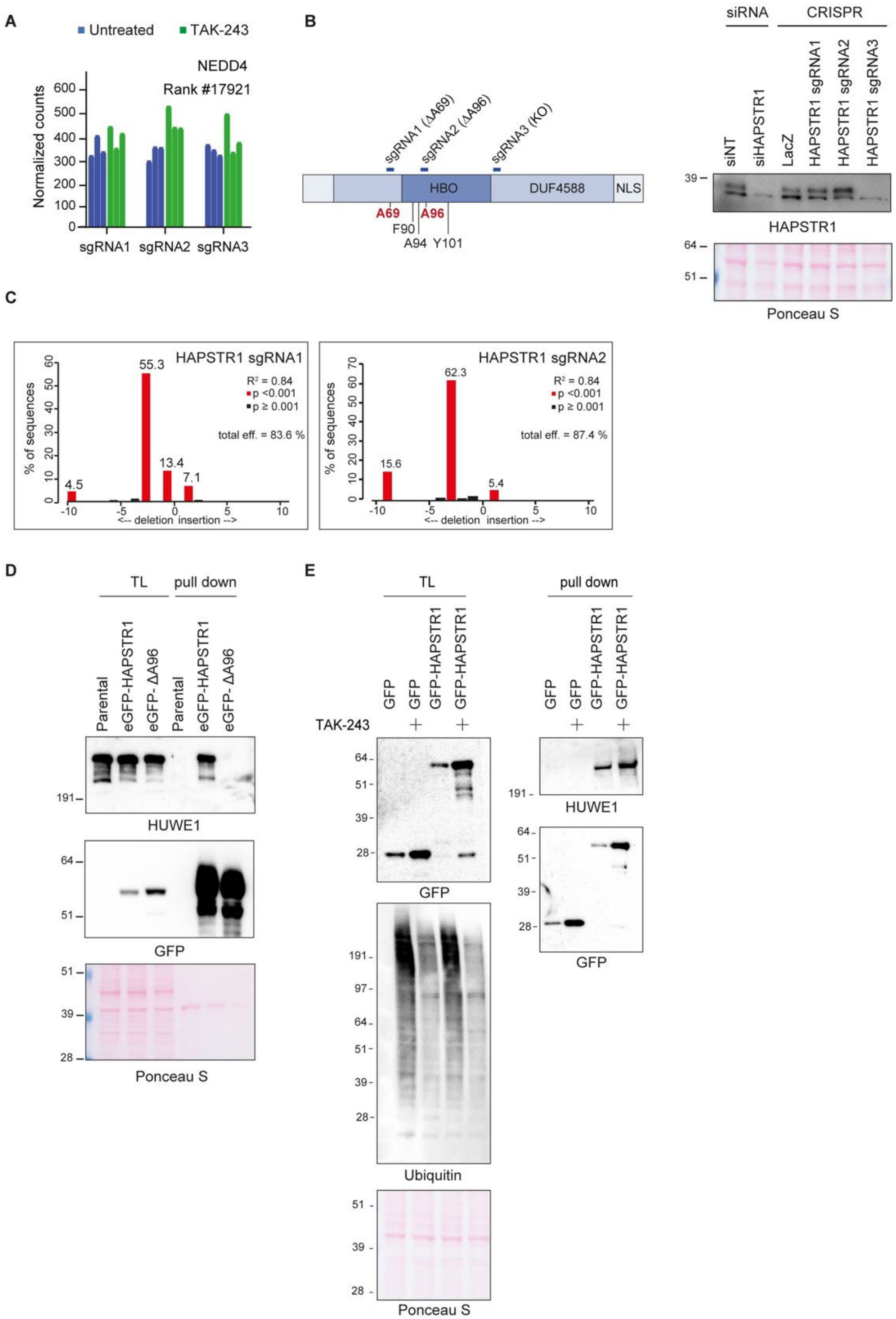
A
Untreated
TAK-243
NEDD4
Rank #17921
Normalized counts
sgRNA1
sgRNA2
sgRNA3
B
sgRNA1 (ΔA69)
sgRNA2 (ΔA96)
sgRNA3 (KO)
HBO
DUF4588
NLS
A69
A96
F90
A94
Y101
siRNA
CRISPR
siNT
siHAPSTR1
LacZ
HAPSTR1 sgRNA1
HAPSTR1 sgRNA2
HAPSTR1 sgRNA3
HAPSTR1
Ponceau S
C
HAPSTR1 sgRNA1
% of sequences
55.3
13.4
7.1
4.5
R² = 0.84
p <0.001
p ≥ 0.001
total eff. = 83.6 %
<-- deletion insertion -->
HAPSTR1 sgRNA2
62.3
15.6
5.4
R² = 0.84
p <0.001
p ≥ 0.001
total eff. = 87.4 %
<-- deletion insertion -->
D
TL
pull down
Parental
eGFP-HAPSTR1
eGFP- ΔA96
HUWE1
GFP
Ponceau S
E
TL
pull down
GFP
GFP-HAPSTR1
TAK-243
HUWE1
GFP
Ubiquitin
Ponceau S

**Figure S3. Analysis of sgRNA-mediated genome editing at the HAPSTR1 locus and interactions with HUWE1 (related to Figures 2 and 3).**

**A** Bar graph of normalized counts for individual sgRNAs targeting the NEDD4 gene from the chemogenetic CRISPR KO screen.

**B** Left: Schematic overview of HAPSTR1 containing a conserved domain of unknown function (DUF4588) and a C-terminal nuclear localization signal (NLS). Depicted are the targeting sequences of the three sgRNA's and the alanine deletions introduced by sgRNA1 (A69) and sgRNA2 (A96) in red. Shown in black are the amino acids whose mutations inhibit HUWE1 binding (24). Right: representative immunoblots of polyclonal cell samples from the MCA assay against endogenous HAPSTR1 (4 days post Cas9 induction) compared to cells transfected with the indicated siRNAs, with Ponceau S as loading control.

**C** Quantitative profile of all insertions and deletions in the HAPSTR1 sgRNA1 and sgRNA2 edited cell pools. For sgRNA1 edited cells, cDNA was extracted and for sgRNA2 edited cells, genomic DNA was used for the TIDE analysis from n=2 independent experiments.

**D** Immunoblot analysis against GFP, HUWE1 and ubiquitin in total lysates (TL) and GFP-trap precipitates from RPE1-p53$^{-/-}$ cells ectopically expressing GFP, GFP-HAPSTR1 or GFP-ΔA69 treated with TAK-243 (0.5 µM, 17h) where indicated. Ponceau S staining was used as loading control. Data shown are representative of n=3 independent experiments.

**E** Immunoblot analysis against GFP, HUWE1 and ubiquitin in total lysates (TL) and GFP-trap precipitates from RPE1-p53$^{-/-}$ cells ectopically expressing GFP or GFP-HAPSTR1 and treated with TAK-243 (0.5 µM, 17h) where indicated. Ponceau S staining was used as loading control. Data shown are representative of n=3 independent experiments.

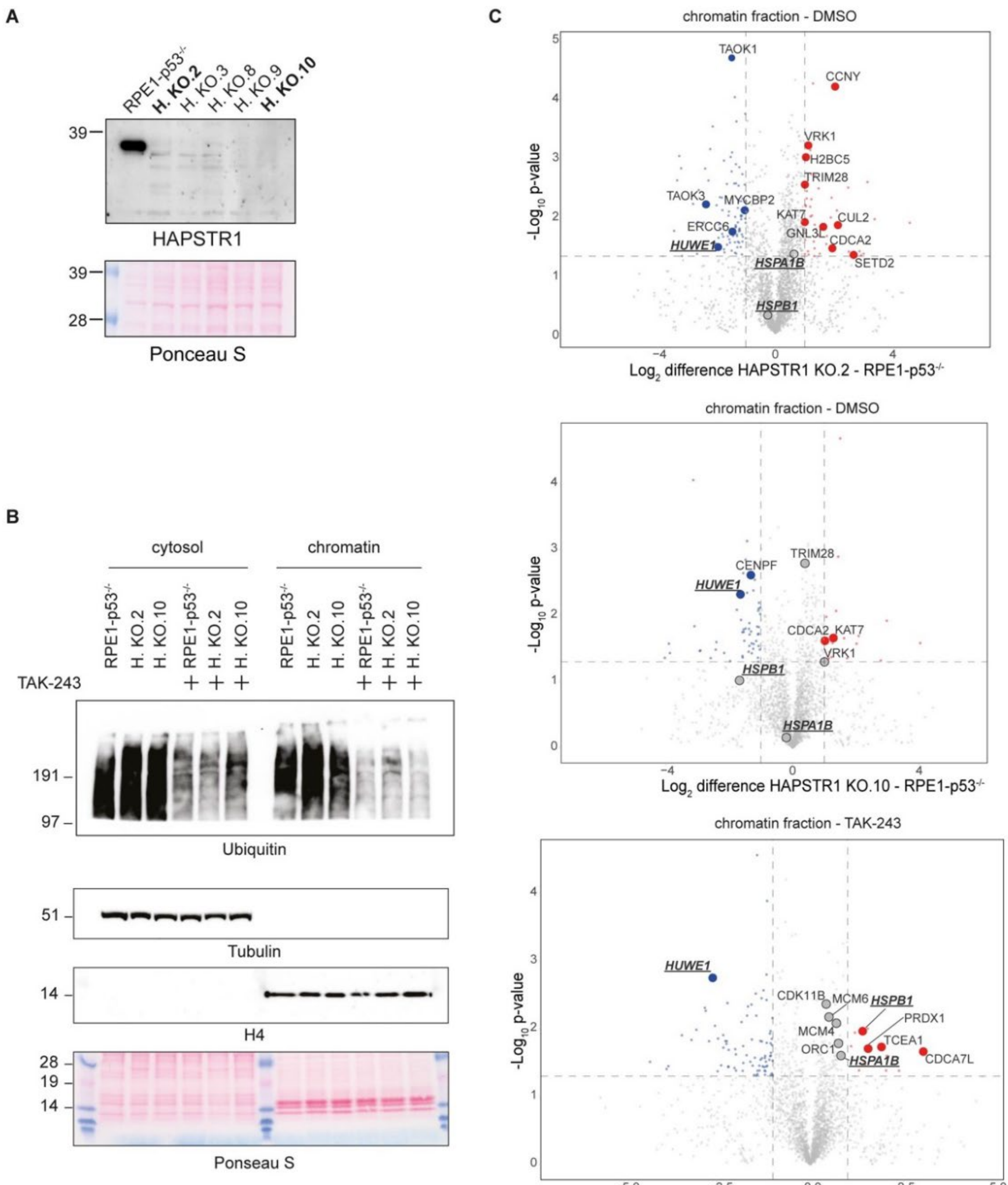


**Figure S4. Analysis of chromatin-associated proteins (related to Figure 4).**

**A** Immunoblots analysis of RPE1-p53-/- parental cells and HAPSTR1-KO clones against endogenous HAPSTR1. Ponceau S: loading control.

**B** Subcellular fractionation in RPE1-p53-/- and HAPSTR1-KO cells allowed for separation of cytosolic and chromatin fractions. Tubulin, histone H4 and ubiquitin were analysed by immunoblotting to confirm subcellular fractionation and the presence of ubiquitin in the chromatin fraction. Ponceau S: loading control.

**C** Effects of HAPSTR1 loss on the chromatin-associated proteome. Volcano plot of proteins enriched in (red) or depleted from (blue) the chromatin fraction of HAPSTR1-KO.2 or HAPSTR1-KO.10 cells compared to RPE1-p53$^{-/-}$ cells treated with DMSO or TAK-243 as indicated. For statistical analysis, FDR was set to 1%. (n=4 independent experiments).

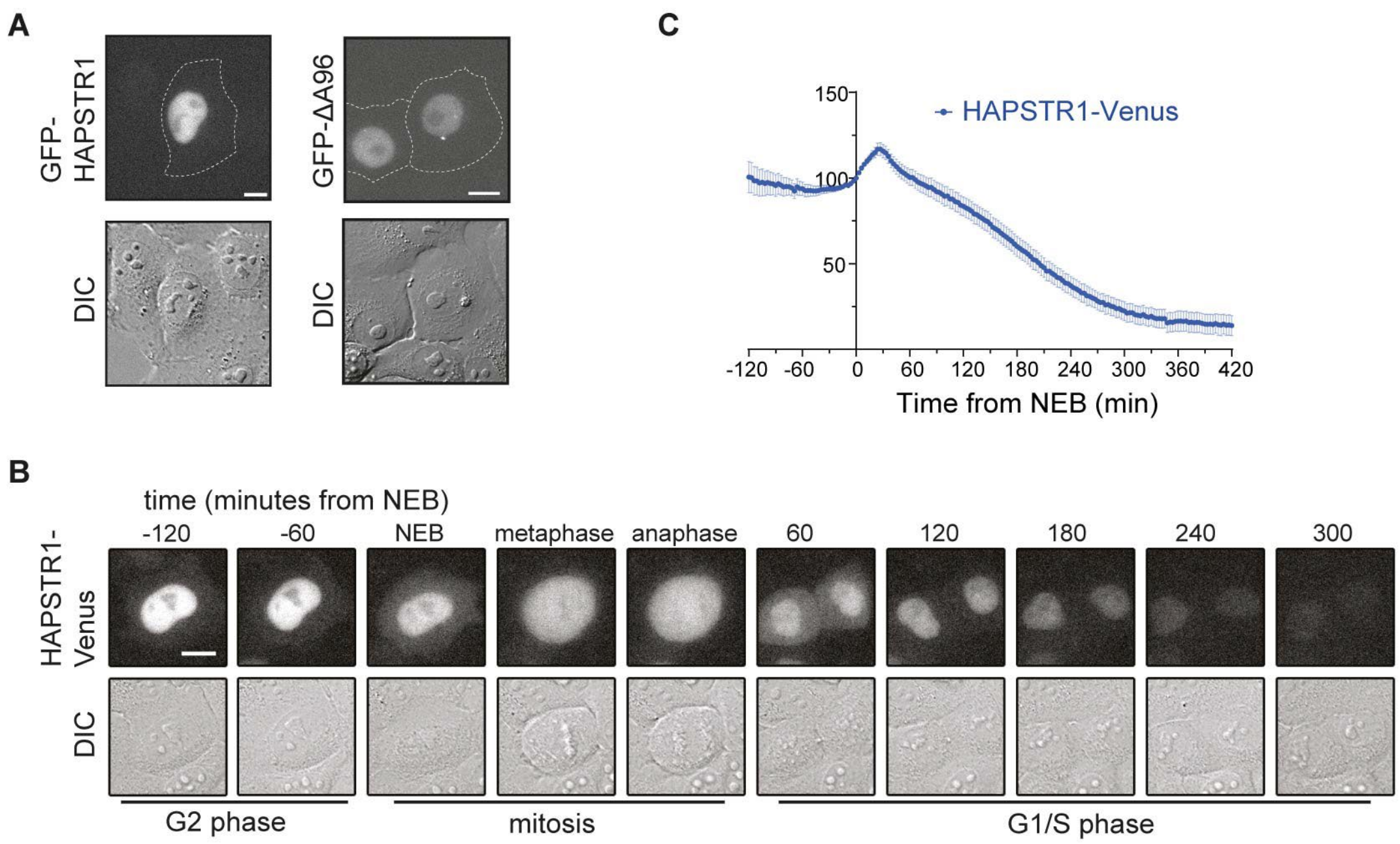


**Figure S5. HAPSTR1 protein is downregulated post mitosis (related to Figure 5).**

**A** Intracellular localization of HAPSTR1 in interphase cells. Representative confocal images of U2OS cells transiently expressing GFP-HAPSTR1 or GFP-HAPSTR1-ΔA96 are shown. Scale bar = 5 µm. The GFP-HAPSTR1 image and corresponding DIC image are intentionally reused from Figure 5A.

**B, C** Downregulation of HAPSTR1 post mitosis. **A** Representative confocal stills from a time-lapse of dividing U2OS cells transiently expressing HAPSTR1-Venus acquired every 3 minutes for 18h. Scale bar = 10 µm. **B** Plot of HAPSTR1-Venus fluorescence intensity (mean ± SEM) against time (min) post nuclear envelope breakdown (NEB, t=0) for 31 cells recorded from n=3 independent experiments.

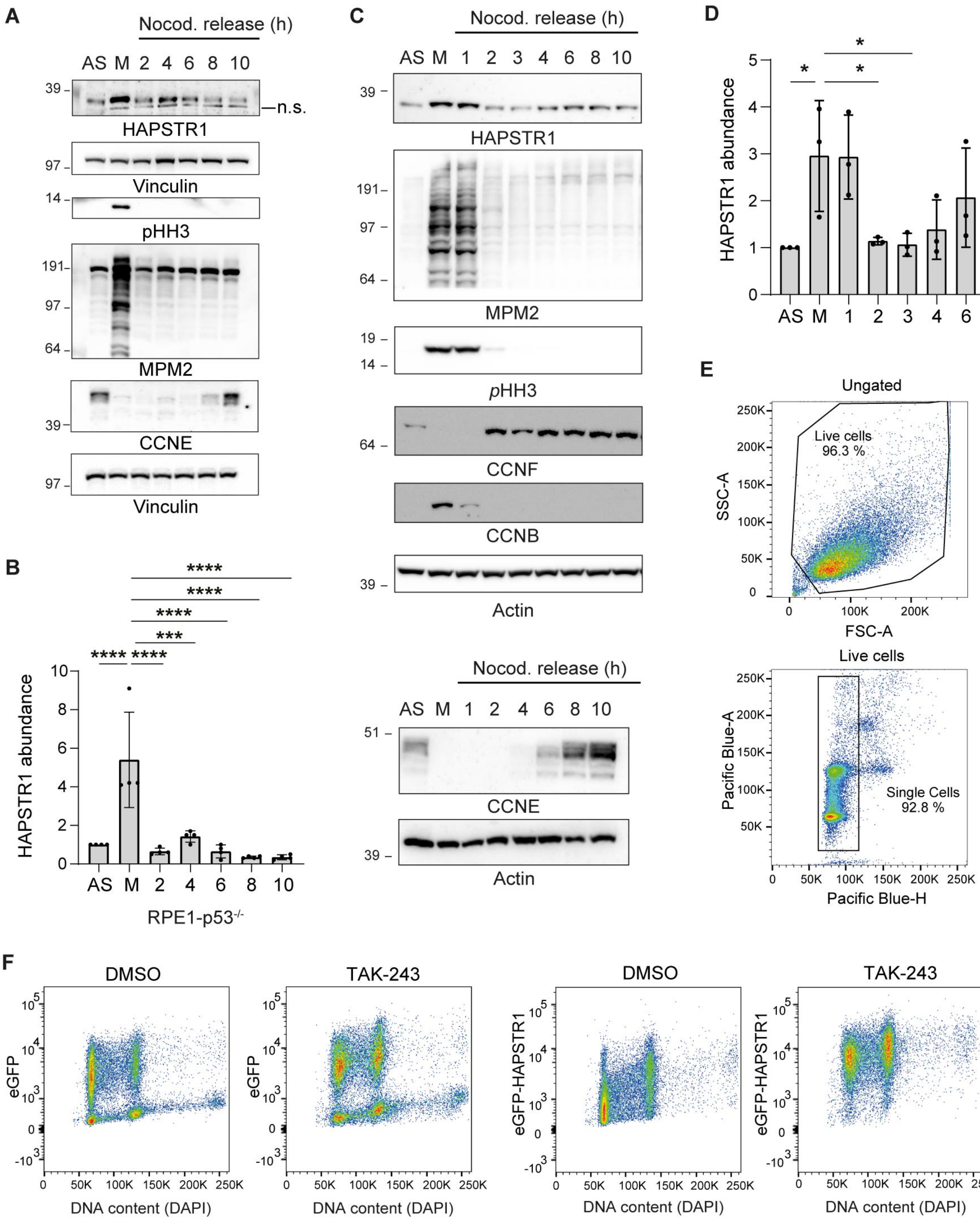
A
Nocod. release (h)
AS M 2 4 6 8 10
39
—n.s.
HAPSTR1
97
Vinculin
14
pHH3
191
97
64
MPM2
39
CCNE
97
Vinculin
B
****
****
****
***
**** ****
HAPSTR1 abundance
10
8
6
4
2
0
AS M 2 4 6 8 10
RPE1-p53-/-
C
Nocod. release (h)
AS M 1 2 3 4 6 8 10
39
HAPSTR1
191
97
64
MPM2
19
14
pHH3
64
CCNF
CCNB
39
Actin
Nocod. release (h)
AS M 1 2 4 6 8 10
51
CCNE
39
Actin
D
*
*
*
HAPSTR1 abundance
5
4
3
2
1
0
AS M 1 2 3 4 6 8 10
E
Ungated
Live cells
96.3 %
SSC-A
FSC-A
250K
200K
150K
100K
50K
0
100K
200K
Live cells
Single Cells
92.8 %
Pacific Blue-A
Pacific Blue-H
0 50K 100K 150K 200K 250K
F
DMSO
TAK-243
DMSO
TAK-243
eGFP
eGFP
eGFP-HAPSTR1
eGFP-HAPSTR1
DNA content (DAPI)
DNA content (DAPI)
DNA content (DAPI)
DNA content (DAPI)
0 50K 100K 150K 200K 250K

**Figure S6. HAPSTR1 protein level dynamics across the cell cycle (related to Figure 5).**
**A-D** Protein level dynamics of endogenous HAPSTR1 during cell cycle progression in **A, B** RPE1-$p53^{-/-}$ and **C, D** HeLa cells. Asynchronous cells (AS) versus those synchronized using nocodazole, harvested by mitotic shake off (M), and released in fresh medium over time (h), were lysed and analyzed by immunoblotting against endogenous HAPSTR1, along with MPM2, phosphorylated histone H3 (pHH3), CCNF, CCNB and CCNE (synchronization controls) and actin or vinculin (loading controls). **A, C** Representative blots from n=3 independent experiments are shown. **B, D** Bar graphs depict HAPSTR1 intensity corrected to the loading control, normalized to AS and expressed relative to the mean of mitotic cells (M) (mean ± SD, one-way ANOVA, *$p<0.05$, ***$p<0.001$, ****$p<0.0001$).
**E, F** Flow cytometry plots showing **E** the gating strategy for live single cells and **F** DNA content (DAPI) analysis as a function of GFP versus GFP-HAPSTR1 fluorescence intensity.

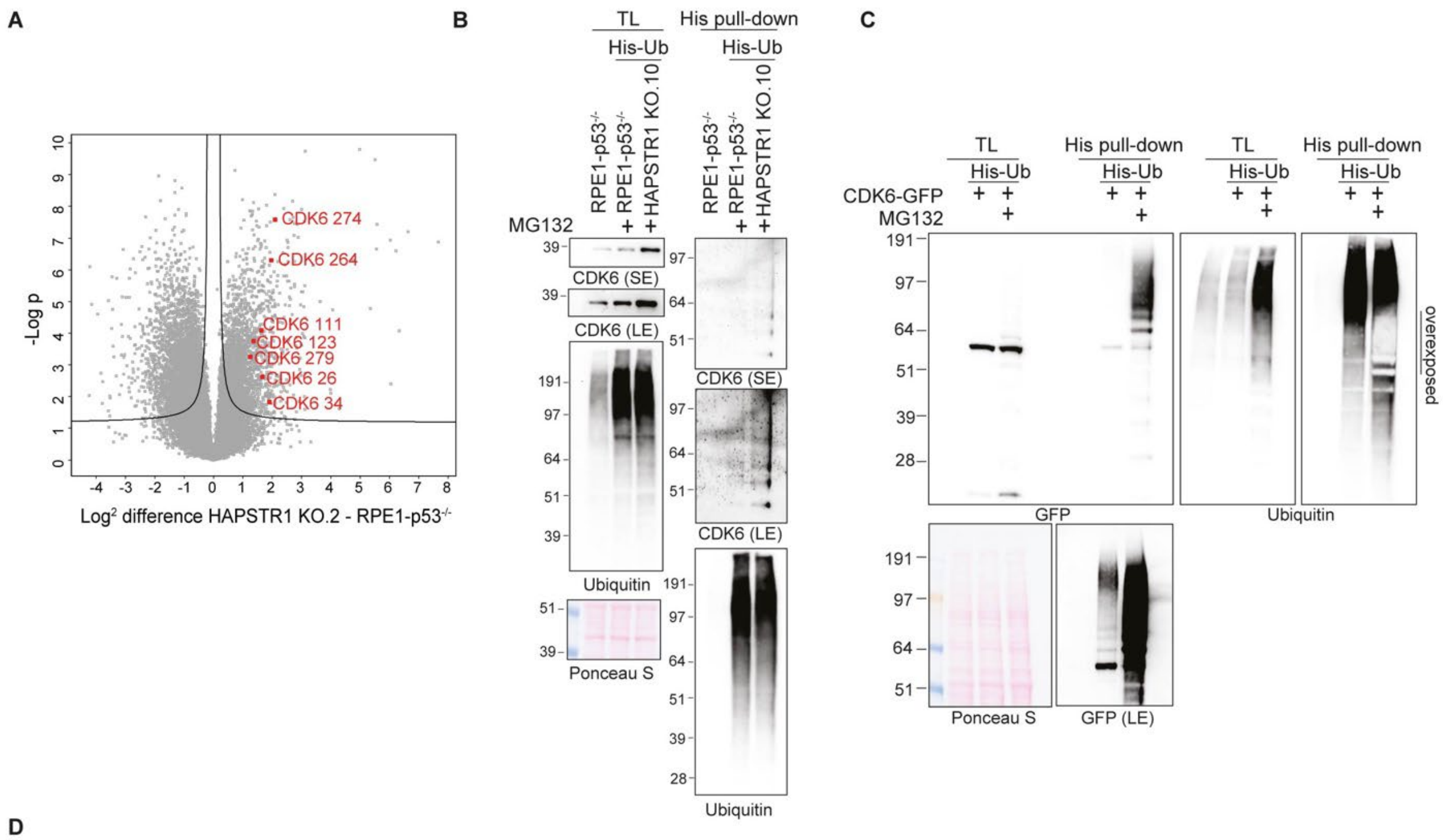


D

| Ub site | RPE1-p53-/- R1 | RPE1-p53-/- R2 | RPE1-p53-/- R3 | RPE1-p53-/- R4 | RPE1-p53-/- R5 | HAPSTR1 KO.2 R1 | HAPSTR1 KO.2 R2 | HAPSTR1 KO.2 R3 | HAPSTR1 KO.2 R4 | HAPSTR1 KO.2 R5 |
|---|---|---|---|---|---|---|---|---|---|---|
| 26 | 50312 | 42103 | 13745 | 27301 | 15748 | 96702 | 69631 | 74851 | 112667 | 90075 |
| 34 | 21302 | 38403 | 38094 | 6469 | 27452 | 99825 | 114363 | 127631 | 31340 | 124414 |
| 111 | 25900 | 25463 | 29255 | 15767 | 25435 | 106314 | 58113 | 63908 | 84832 | 76082 |
| 123 | 10857 | 7504 | 15114 | 13121 | 14962 | 30050 | 29675 | 33776 | 26641 | 42218 |
| 264 | 52380 | 50383 | 59283 | 48212 | 73936 | 260216 | 189450 | 224298 | 235427 | 244151 |
| 274 | 254574 | 249937 | 253228 | 252669 | 299264 | 1137267 | 946682 | 1213411 | 1155324 | 1400139 |
| 279 | 134619 | 124512 | 123303 | 213937 | 101244 | 234819 | 385179 | 392026 | 316260 | 385090 |

**Figure S7. Elevated ubiquitination of CDK6 in cells lacking HAPSTR1 (related to Figure 6).**
**A** Effect of HAPSTR1 ablation on ubiquitination of CDK6. Volcano plot of endogenous ubiquitin substrates in HAPSTR1-KO.2 versus RPE1-p53$^{-/-}$ cells, highlighting all detected ubiquitination sites of CDK6.

**B, C** Ubiquitination of CDK6 analysed by Ni-NTA bead capture from cells expressing His10-Ub. Immunoblot analyses of His10-Ub precipitates for **B** endogenous CDK6 from RPE1-p53$^{-/-}$ versus HAPSTR1-KO cells or **C** ectopically expressed CDK6-GFP from cells treated in the absence or presence of MG132 (10 µM, 6h) as indicated. Ponceau S: loading control. Representative data for n=3 independent experiments are shown.

**D** Peptide intensities of endogenous ubiquitinated CDK6 peptides identified by UbiSite. Colour coding reflects relative lower (blue) and higher (red) intensity values.

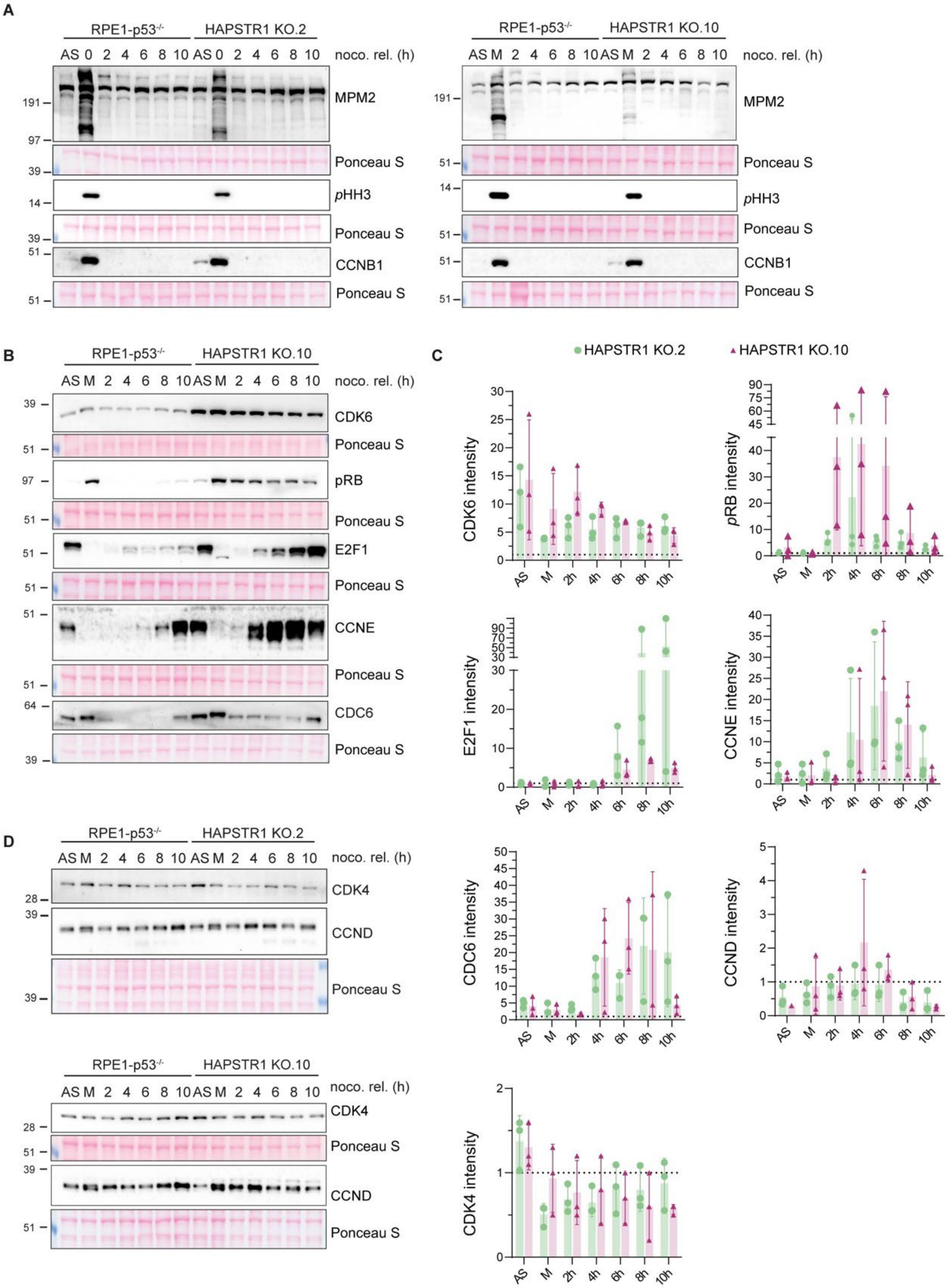
A
RPE1-p53-/-
HAPSTR1 KO.2
AS 0 2 4 6 8 10 AS 0 2 4 6 8 10
noco. rel. (h)
MPM2
Ponceau S
pHH3
CCNB1
HAPSTR1 KO.10
AS M 2 4 6 8 10 AS M 2 4 6 8 10
B
RPE1-p53-/-
HAPSTR1 KO.10
CDK6
pRB
E2F1
CCNE
CDC6
C
HAPSTR1 KO.2
HAPSTR1 KO.10
CDK6 intensity
pRB intensity
E2F1 intensity
CCNE intensity
CDC6 intensity
CCND intensity
CDK4 intensity
AS M 2h 4h 6h 8h 10h
D
RPE1-p53-/-
HAPSTR1 KO.2
HAPSTR1 KO.10
CDK4
CCND
Ponceau S

**Figure S8. Hyperactivation of the CDK6 pathway in the absence of HAPSTR1 (related to Figure 6).**

**A** Controls for nocodazole synchronization and release of RPE1-p53$^{-/-}$ and HAPSTR1-KO cells. Immunoblot analyses of MPM2 and phosphorylated (pHH3) confirm synchronization of mitotic samples (M) and loss of cyclin-B1 (CCNB1) demonstrates mitotic exit. Ponceau S: loading control. Representative blots of n=3 independent experiments.

**B-D** Effects of HAPSTR1 ablation on protein abundance of CDK6, CDK4, functionally related cyclins and downstream pathway targets in mitosis and early G1. Immunoblot analysis of **B** CDK6, CDC6, CCNE, pRB, and **D** E2F1 or D CDK4 and CCND in RPE1-p53$^{-/-}$ and HAPSTR1-KO.10 cells synchronized at mitosis (M) and released over time (h), compared to asynchronous cells (AS). Representative data are shown. **C** Quantification of immunoblot intensities for the indicated endogenous proteins in HAPSTR1-KO.2 and HAPSTR1-KO.10 cell lines (mean ± SD) expressed relative to RPE1-p53$^{-/-}$ (1.0 marked by dashed lines) from n=3 independent experiments.

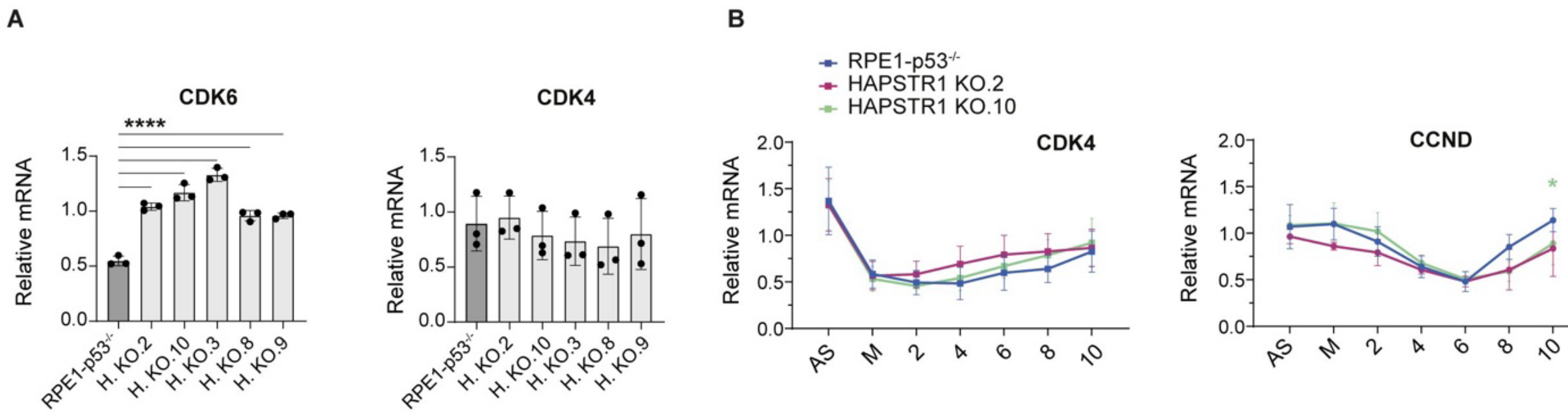


**Figure S9. Transcriptional upregulation of the CDK6 pathway in the absence of HAPSTR1 (related to Figure 6).**

**A** Effects of HAPSTR1 ablation on transcription of CDK6 (left) and CDK4 (right). RT-qPCR analysis of CDK6 and CDK4 mRNA levels in RPE1-p53$^{-/-}$ and HAPSTR1-KO asynchronous cell lines normalized to housekeeping genes (ARP, CAPNS1, RPS11, SRPR and TBP). Bar graphs report on mean ± SD from n=3 biological replicates. Statistics assessed using Dunnett's One-Way ANOVA, ****$p<0.0001$.

**B** Effects of HAPSTR1 ablation on transcriptional dynamics of CDK4 and CCND from mitosis to early G1. RT-qPCR analysis of CDK4 (left) and CCND (right) mRNA levels in RPE1-p53$^{-/-}$ (blue), HAPSTR1-KO.2 (magenta) and HAPSTR1-KO.10 (green) cells synchronized in mitosis (M) using nocodazole block and released over time (h) normalized to housekeeping genes ARP, CAPNS1, RPS11, SRPR and TBP. The graphs depict the normalized mRNA expression of 4 independent experiments, mean ± SD, two-way ANOVA followed by Tukey's multiple comparison test, *$p<0.05$.

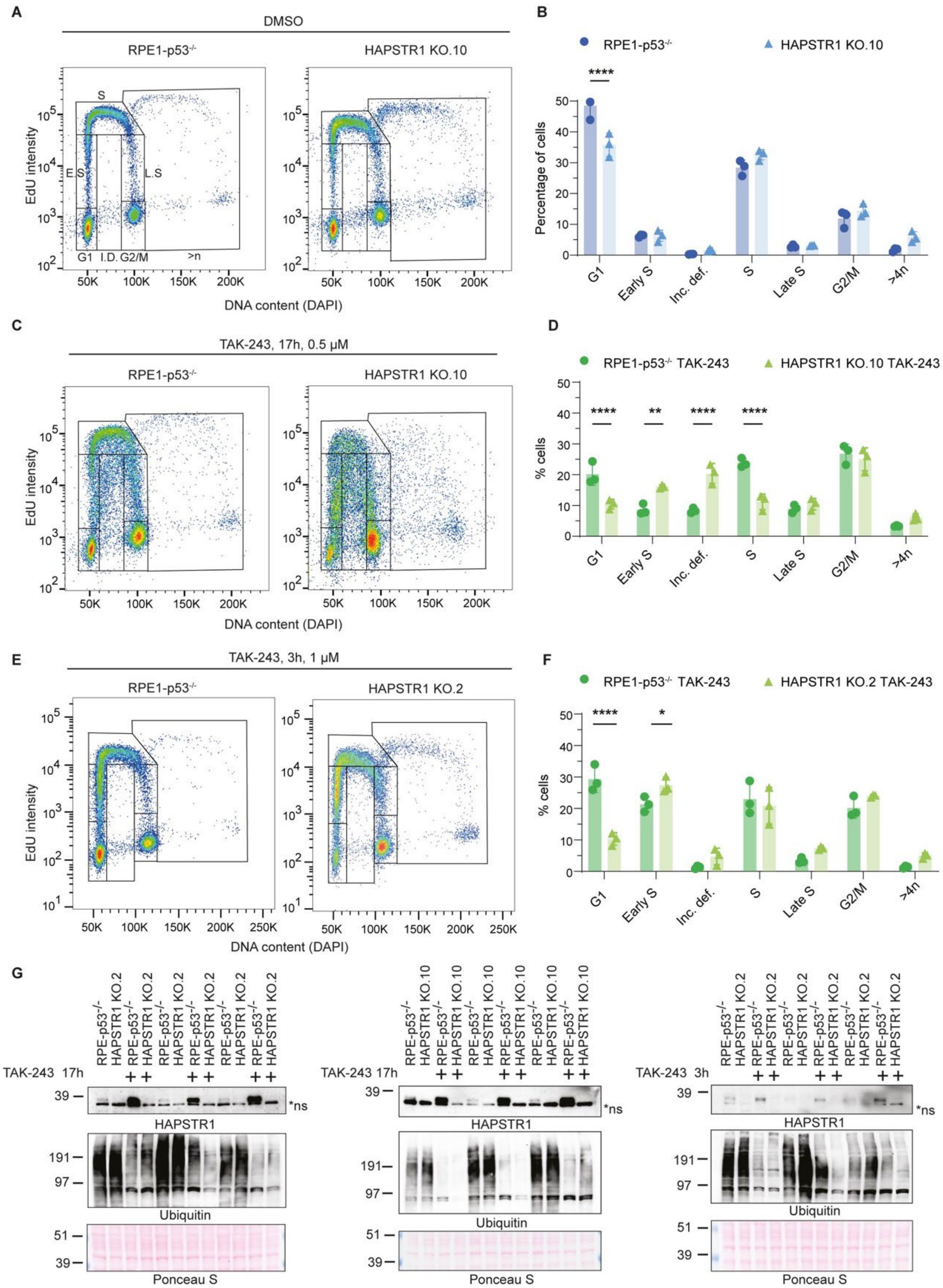

A
DMSO
RPE1-p53-/-
HAPSTR1 KO.10
EdU intensity
DNA content (DAPI)
S
E.S
L.S
G1
I.D.
G2/M
>n
B
RPE1-p53-/-
HAPSTR1 KO.10
Percentage of cells
G1
Early S
Inc. def.
S
Late S
G2/M
>4n
C
TAK-243, 17h, 0.5 µM
RPE1-p53-/-
HAPSTR1 KO.10
D
RPE1-p53-/- TAK-243
HAPSTR1 KO.10 TAK-243
% cells
E
TAK-243, 3h, 1 µM
RPE1-p53-/-
HAPSTR1 KO.2
F
RPE1-p53-/- TAK-243
HAPSTR1 KO.2 TAK-243
G
TAK-243 17h
TAK-243 3h
HAPSTR1
Ubiquitin
Ponceau S
*ns

**Figure S10. HAPSTR1 regulates cell cycle progression under ubiquitin signalling blockade (related to Figure 7).**

**A-F** Effects of HAPSTR1 ablation on cell cycle progression. Representative flow cytometry analyses of DNA content (DAPI) and 5-Ethynyl-2′-deoxyuridine (EdU) incorporation (DNA replication) in RPE1-p53$^{-/-}$ versus HAPSTR1-KO.10 cells, treated with **A** DMSO, **C** TAK-243 (17h, 0.5 μM) or **E** TAK-243 (3h, 1.0 μM) and pulsed with EdU are shown. Quantifications of population percentages per cell cycle phase (indicated by gates set in the plots) for **B** DMSO (blue) and **D, F** TAK-243 (green) conditions from n=3 independent experiments (mean ± SD, two-way ANOVA followed by Tukey's multiple comparison test, *$p<0.05$, **$p<0.01$, ***$p<0.001$, ****$p<0.0001$). Cell cycle phases: G1, S, G2, M; E.S.: early S phase, L.S.: late S phase, I.D. and Inc. def.: incorporation defects.

**G** Representative immunoblot analyses for HAPSTR1 and ubiquitin in RPE1-p53$^{-/-}$ and HAPSTR1-KO cells treated with DMSO or TAK-243 as indicated. Ponceau S: loading control.

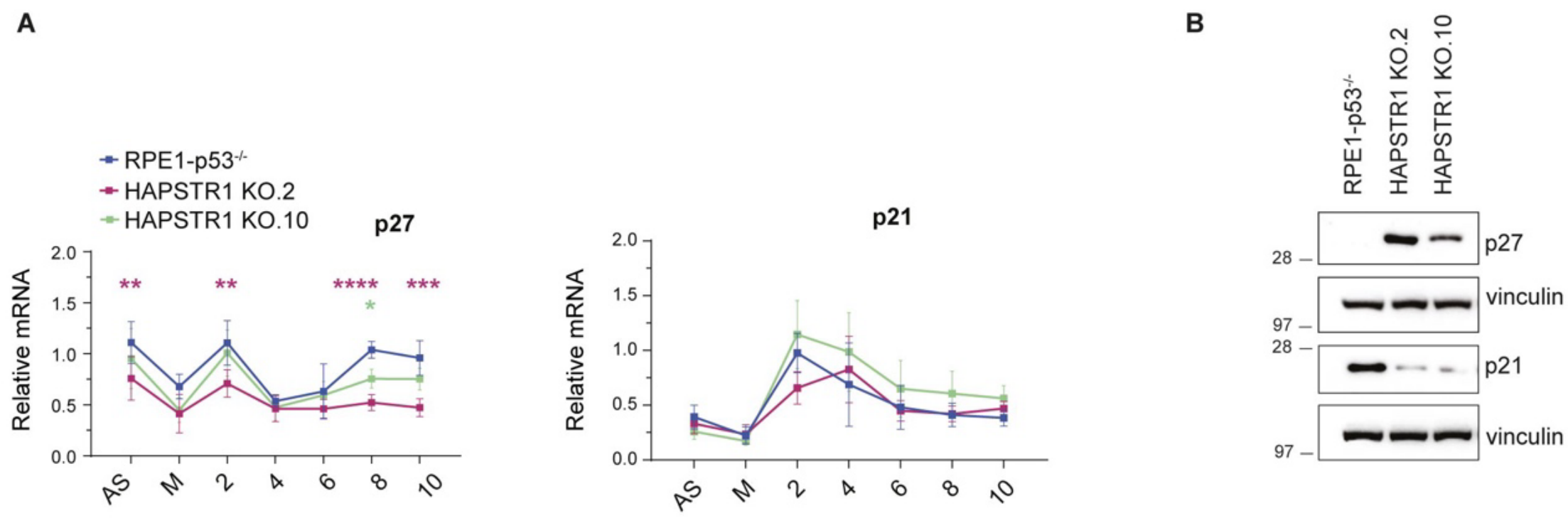


**Figure S11. HAPSTR1 upregulates p27 and p21 (related to Figure 7).**

**A** Immunoblotting of p27 and p21 in RPE1-p53$^{-/-}$ and HAPSTR1-KO cells, with Vinculin as loading control. Data shown are representative of n=3 independent experiments.

**B** mRNA expression of p27 and p21 assessed by RT-qPCR. The graphs depict the normalized mRNA expression of 4 independent experiments (mean ± SD, two-way ANOVA followed by Tukey's multiple comparison test, $*p<0.05$ $**p<0.01$, $***p<0.001$, $****p<0.0001$.

**Table S1. Genome editing of the HAPSTR1 locus (related to Figure 2).**
Detailed results of the genomic editing of the HAPSTR1 locus.

| Experiment | Cell line | sgRNA transduction | sgRNA sequence | total editing efficiency | % of sequence with 3 bp deletion | genomic DNA or cDNA |
|---|---|---|---|---|---|---|
| Multi-colour competition assay (related to Figure 2, S3), polyclonal CRISPR KO cells | RPE1 TP53-/- TetOn-cas9 | sgRNA 1 C16orf72 | GTAGAGCTGG GCCACGGCGG | 86.00% | 63.30% | cDNA |
| | | sgRNA 2 C16orf72 | GTAGAGATTG GTGACGGCGG | 88% | 63.90% | genomic DNA |
| | | sgRNA 3 C16orf72 | GGAGCTCTAG AGTTTCGTGG | 53.60% | | genomic DNA |
| | | sgRNA 1 LacZ | CCCGAATCTCT ATCGTGCGG | | | |
| Generation of single knock-out clones | RPE1 TP53-/- TetOn-cas9 HAPSTR1 KO.2 | sgRNA 2 C16orf72 | GTAGAGATTG GTGACGGCGG | | | |
| | RPE1 TP53-/- TetOn-cas9 HAPSTR1 KO.3, 8, 9, 10 | sgRNA 3 C16orf72 | GGAGCTCTAG AGTTTCGTGG | | | |

## Table S2. Key resources.

| Antibody | Source | Identifier |
|---|---|---|
| Mouse-anti-Ubiquitin, 1:1000 | Santa Cruz Biotechnology | sc-8017, RRID:AB_628423 |
| Mouse-anti-CAS9, 1:1000 | Gift from AG Jochemsen | N/A |
| Mouse-anti-P53 (DO-1), 1:1000 | Santa Cruz Biotechnology | sc-126, RRID:AB_628082 |
| Rabbit-anti-CDK1, 1:5000 | Gift from Michele Pagano | N/A |
| Rabbit-anti-C16ORF72, 1:500 | Atlas Antibodies | hpa067492, RRID:AB_2685850 |
| Rabbit-anti-CDK4, 1:1000 | Bethyl | A304-224A, RRID:AB_2620421 |
| Mouse-anti-MPM2, 1:1000 | Millipore | 05-368, RRID:AB_309698 |
| Mouse-anti-Vinculin, 1:5000 | Gift from AG Jochemsen | N/A |
| Rabbit-anti-CDK6, 1:1000 | Cell Signalling Technology | 13331, RRID:AB_2721897 |
| Rabbit-anti-*p*RB, 1:5000 | Cell Signalling Technology | 8516, RRID:AB_11178658 |
| Mouse-anti-E2F1, 1:500 | Thermo Fisher | 32-1400, RRID:AB_2533065 |
| Rabbit-anti-*p*HH3, 1:5000 | Bethyl | A301-844, RRID:AB_1264320 |
| Mouse-anti-CCNE, 1:500 | Santa Cruz BT | Sc-247, RRID:AB_627357 |
| Mouse-anti-CCND1, 1:500 | Santa Cruz BT | Sc-20044, RRID:AB_627346 |
| Rabbit-anti-HUWE1, 1:1000 | Bethyl Laboratories | A300-486A, RRID:AB_2264590 |
| Mouse-anti-GFP, 1:5000 | Roche | 11814460001, RRID:AB_390913 |
| Rabbit-anti-V5, 1:5000 | Abcam | ab9116, RRID:AB_307024 |
| Mouse-anti-Actin, 1:5000 | Sigma-Aldrich | A5441, RRID:AB_476744 |
| Rabbit-anti-CCNB1 | Cell Signaling Technology | 4138, RRID:AB_2072132 |
| Rabbit-anti-CDC6 | Abcam | ab109315, RRID:AB_10862545 |
| Rat-anti-BrdU, 1:200, recognizes CIdU | Abcam | Clone BU1/75, RRID:AB_305426 |
| Mouse-anti-BrdU, 1:100, recognizes IdU | BD Biosciences | clone B44, PRID:AB_400326 |
| Mouse-anti-HSF1, 1:1000 | Abcam | ab76076, RRID:AB_1310328 |
| Mouse-anti-HSPB1, 1:1000 | Santa Cruz BT | sc-13132, RRID:AB_627755 |
| Mouse-anti-γH2AX phosphoS139 clone JBW301, 1:1000 | Sigma-Aldrich | 05-636-I, RRID:AB_2755003 |
| Goat-anti-Mouse Alexa Fluor 555, 1:1000 | Thermo Fisher Scientific | A-21424, RRID:AB_141780 |
| Goat-anti-Rat Alexa Fluor 555, 1:1000 | Thermo Fisher Scientific | A-21434, RRID:AB_2535855 |
| Goat-anti-Mouse Alexa Fluor 488, 1:1000 | Thermo Fisher Scientific | A-11029, RRID:AB_2534088 |
| Mous-anti-p27, 1:1000 | Santa Cruz BT | Sc-1641, RRID:AB_628074 |
| Rabbit-anti-p21, 1:1000 | Santa Cruz BT | Sc-397, RRID:AB_632126 |
| | | |
| **Oligonucleotides** | **Source** | **Identifier** |
| Site-directed mutagenesis to generate HAPSTR1-ΔA96 fw: GTCACCAATCTCTACAAAGAAAGC | This study | N/A |
| Site-directed mutagenesis to generate HAPSTR1-ΔA96 rv: GGTGGCTGCGTTTTGGAA | This study | N/A |
| CAPNS1 fw: ATGGTTTTGGCATTGACACATG | Gift from AG Jochemsen | N/A |
| CAPNS1 rv: GCTTGCCTGTGGTGTCGC | Gift from AG Jochemsen | N/A |
| TBP fw: CGGCTGTTTAACTTCGCTTC | Gift from AG Jochemsen | N/A |
| TBP rv: CACACGCCAAGAAACAGTGA | Gift from AG Jochemsen | N/A |
| SRPR fw: CATTGCTTTTGCACGTAACCAA | Gift from AG Jochemsen | N/A |
| SRPR rv: ATTGTCTTGCATGCGGCC | Gift from AG Jochemsen | N/A |

| | | |
|---|---|---|
| RPS11 fw: AAGCAGCCGACCATCTTTCA | Zuidervaart et al. (81) | N/A |
| RPS11 rv: CGGGAGCTTCTCCTTGCC | Zuidervaart et al. (81) | N/A |
| HAPSTR1 fw: CCTAACCGAGCTACTTCAACGG | Origene | HP210639 |
| HAPSTR1 rv: CGCACGCTTATACTAGCCATTGC | Origene | HP210639 |
| CDK4 qPCR fw: CCATCAGCACAGTTCGTGAGGT | Origene | HP200061 |
| CDK4 qPCR rv: TCAGTTCGGGATGTGGCACAGA | Origene | HP200061 |
| CDK6 qPCR fw: GGATAAAGTTCCAGAGCCTGGAG | Origene | HP205182 |
| CDK6 qPCR rv: GCGATGCACTACTCGGTGTGAA | Origene | HP205182 |
| cyclin D1 qPCR fw: TCTACACCGACAACTCCATCCG | Origene | HP216596 |
| cyclin D1 qPCR rv: TCTGGCATTTTGGAGAGGAAGTG | Origene | HP216596 |
| cyclin E1 qPCR fw: TGTGTCCTGGATGTTGACTGCC | Origene | HP205164 |
| cyclin E1 qPCR rv: CTCTATGTCGCACCACTGATACC | Origene | HP205164 |
| E2F1 qPCR fw: GGACCTGGAAACTGACCATCAG | Origene | HP208401 |
| E2F1 qPCR rv: CAGTGAGGTCTCATAGCGTGAC | Origene | HP208401 |
| p27 qPCR fw: ATAAGGAAGCGACCTGCAACCG | Origene | HP207435 |
| p27 qPCR rv: TTCTTGGGCGTCTGCTCCACAG | Origene | HP207435 |
| p21 qPCR fw: AGCAGAGGAAGACCATGTGGA | Gift from AG Jochemsen | N/A |
| p21 qPCR rv: AATCTGTCATGCTGGTCTGCC | Gift from AG Jochemsen | N/A |
| CDC6 qPCR fw: GGAGATGTTCGCAAAGCACTGG | Origene | HP205178 |
| CDC6 qPCR rv: GGAATCAGAGGCTCAGAAGGTG | Origene | HP205178 |
| SMARTpool ON-TARGETplus human C16orf72 | Dharmacon | L-031072-00-0020 |
| SMARTpool ON-TARGETplus human HUWE1 | Dharmacon | L-007185-00-0050 |
| SMARTpool ON-TARGETplus non-targeting | Dharmacon | D-001810-10-50 |
| | | |
| **Chemicals** | **Source** | **Identifier** |
| EdU | Sigma-Aldrich | 900584 |
| Doxycycline | Sigma-Aldrich | D9891-1g |
| TAK-243 | Active Biochem | A-1384 |
| MG132 | Sigma-Aldrich | C2211-5MG |
| ML-792 | MedKoo | 407886 |
| Thymidine | Sigma-Aldrich | T1895-5G |
| Nocodazole | Sigma-Aldrich | M1404-10MG |
| AF647-Picolyl-Azide | Jena Bioscience | CLK-1300-1 |
| CIdU | Merck | C6891 |
| IdU | Merck | I7125 |
| | | |
| **Recombinant DNA** | **Source** | **Identifier** |
| pLentiGuide-NLS-GFP-P2A-Puro | Noordermeer et al. (60) | N/A |
| pLentiGuide-NLS-mCherry-P2A-Puro | Noordermeer et al. (60) | N/A |
| HAPSTR1 cDNA | Sigma-Aldrich | Clone ID 03065 |
| pCW57.1 | Addgene | 41393 |
| His10-ubiquitin-IRES-Puromycin | Cuijpers et al. (61) | N/A |
| pLCKO-TKOv3 CRIPSR guide library | Addgene | 125517 |

| | | |
|---|---|---|
| NLS-GFP | J.J.C. Neefjes | N/A |
| | | |
| **Software** | **Source** | **Version** |
| ImageJ | Schneider et al. (69) | V1.53t |
| Graphpad Prism | GraphPad software | v9.3.1 |
| FlowJo | BD Biosciences | V10.8.1 |
| Cytoscape | Cytoscape Consortium | V3.10.1 |
| Perseus | Max Planck Institute of Biochemistry | V2.0.7.0 |
| MaxQuant | Max Planck Institute of Biochemistry | v.2.1.3.0 |
| Spectronaut | Biognosys | version 18.1.230626.50606 |
| Fastp | https://github.com/OpenGene/fastp | V0.20.1 |
| Proteome Discoverer | Thermo Electron | version 3.2 |

**Data S1. Chemogenetic CRISPR knock-out screen reveals critical regulators within the ubiquitin signalling network essential for cell proliferation (related to Figure 1).**

Gene-by-gene results of the chemogenetic CRISPR KO screen.

**Data S2. HAPSTR1 interactome analysis (related to Figure 3).**

Protein-by-protein results of GFP-Trap co-precipitates with GFP, GFP-HAPSTR1 and GFP-ΔA96 treated in the presence or absence of TAK-243.

**Data S3. Chromatin-associated proteome changes as a function of HAPSTR1 ablation (related to Figure 4).**

Protein-by-protein results of the chromatin-associated proteome changes due to HAPSTR1 ablation.

**Data S4. Transcriptome-wide profiling of transcriptional changes as a function of HAPSTR1 ablation (related to Figure 4).**

Transcript-by-transcript results of the transcriptional changes due to HAPSTR1 ablation.

**Data S5. Proteome-wide profiling of cellular ubiquitination landscape as a function of HAPSTR1 ablation (related to Figure 6).**

Protein-by-protein results of changes in the ubiquitinome due to HAPSTR1 ablation.
